# Evaluation of EEG pre-processing and source localization in ecological research

**DOI:** 10.3389/fnimg.2025.1479569

**Published:** 2025-03-31

**Authors:** Carlos Gomez-Tapia, Bojan Bozic, Luca Longo

**Affiliations:** Artificial Intelligence and Cognitive Load Research Lab, Applied Intelligence Research Centre, School of Computer Science, Technological University Dublin, Dublin, Ireland

**Keywords:** electroencephalography, source localization, ecological settings, inverse modeling, source imaging, eLORETA, pipeline

## Abstract

**Introduction:**

Electroencephalography (EEG) source localization (SL) has shown potential for various applications, from epilepsy and seizure focus localization to psychiatric disorder evaluation. However, questions remain about its neurophysiological plausibility in real-world settings where only EEG signals are available without subject-specific anatomical information. This study investigates whether established pre-processing and source localization methods can produce neurophysiologically plausible activation patterns when applied to naturalistic EEG data without structural magnetic resonance imaging (MRI) or digitized electrode positions.

**Methods:**

Proven methods are aggregated into an end-to-end pipeline that includes automatic pre-processing, eLORETA for source estimation, and a shared forward model derived from the ICBM 2009c Nonlinear Symmetric template and its corresponding CerebrA atlas. The pipeline is validated using two distinct datasets: the Healthy Brain Network (HBN) dataset comparing resting and naturalistic video-watching states and the multi-session and multi-task EEG cognitive dataset (COGBCI) comparing different cognitive workload levels. The validation approach focuses on whether the reconstructed source activations exhibit expected neurophysiological patterns via permutation testing.

**Results:**

Findings revealed significant differences between resting state and video-watching tasks, with greater activation in posterior regions during video-watching, consistent with known visual processing pathways. The cognitive workload analysis similarly showed progressive activation increases with task difficulty, mapping to regions associated with executive function.

**Discussion:**

These results prove that established source localization methods can produce neurophysiologically plausible activation patterns without subject-specific information, highlighting the strengths and limitations of applying these methods to mid-length naturalistic EEG data. This research demonstrates the viability of template-based source analysis for research settings where individual structural imaging is unavailable or impractical.

## 1 Introduction

Electroencephalography (EEG) is a method for non-invasive recording of the brain's electrical activity. Although possessing an excellent temporal resolution, low spatial resolution limits EEG, making precise neural source identification challenging. Over the past five decades, researchers have developed numerous source localization methods to address this limitation. Source localization enhances EEG's spatial resolution, bridging the gap between its excellent temporal resolution and its traditionally limited spatial accuracy. By pinpointing the origins of brain-specific electrical activities, researchers and clinicians can gain deeper insights into the neural underpinnings of cognitive processes, behaviors, and disorders such as Alzheimer's (Aghajani et al., 2013), depression (Zhu et al., 2018), and focal epilepsy (Plummer et al., 2008), among others. Traditional approaches for developing and testing source localization algorithms include averaged trials based on events (Event Related Potentials) (Slotnick, 2004; Tsolaki et al., 2015), integration with fMRI or other neuroimaging modalities (Ritter and Villringer, 2006; Huster et al., 2012), simulations (Supek and Aine, 1993; Yao and Dewald, 2005; Dümpelmann et al., 2009), and intracranial EEG recordings (Bénar et al., 2006; Plummer et al., 2008; Michel and Brunet, 2019). However, a critical gap remains in validating these methods, as the controlled laboratory settings of traditional EEG studies limit their applicability in real-world settings (Alday, 2019). While the efficacy of source localization has been extensively examined in controlled settings, the performance of unimodal EEG source localization techniques with mid-length un-epoched recordings remains underexplored.

This study aims to bridge this gap by investigating whether accurate EEG source localization can be achieved without subject-specific information such as structural MRI or digitized electrode positions. In particular, the research question this study aims to answer is the following:

**How can established pre-processing and source localization methods, when aggregated, produce neurophysiologically plausible activation patterns when evaluated in naturalistic settings without subject-specific information?**

An end-to-end aggregated pipeline for EEG pre-processing and Source Localization suitable for real single-trial (non-averaged) EEG data is designed, developed, and evaluated to address these questions. The proposed approach utilizes unimodal EEG data, emulating real-world settings where subject-specific information might not be available. The aggregated pipeline includes an automatic EEG signal pre-processing pipeline based on Makoto's guidelines (Makoto, 2024), the adoption of eLORETA (Pascual-Marqui, 2007) as a source localization algorithm and a shared forward model derived from the ICBM 2009c Nonlinear Symmetric template (Fonov et al., 2009) and the CerebrA atlas (Manera et al., 2019). Our contribution lies in providing evidence that these methods produce neurophysiologically plausible results even without subject-specific information.

Accurately validating the accuracy of source localization methods without a ground truth is not trivial, given the problem's ill-posed nature. The proposed evaluation procedure compares differences in source space amplitudes for different tasks via permutation testing. Methods are validated using data from 2 different datasets, namely the Healthy Brain Network (HBN) dataset (Alexander et al., 2017) and the multi-session and multi-task EEG cognitive dataset for passive brain-computer interfaces (COGBCI) datasets (Hinss et al., 2023). Results demonstrate EEG task variability at the sensor space is retained at the source space level. Paired permutation testing shows a significant task-based difference between source space amplitudes both on the overall source space and in individual brain regions. These results validate the aggregated pipeline's effectiveness in retaining informative features under the self-imposed constraint of not having access to subject-specific information.

While individual anatomical variations can influence source localization accuracy, standardized head models remain viable for many research applications. Our validation across two independent datasets demonstrates consistent and interpretable activation patterns, suggesting that template-based source localization can effectively capture meaningful neural activity patterns. This finding aligns with previous research (Fuchs et al., 2002; Valdés-Hernández et al., 2009; Song et al., 2015), which showed that template-based approaches could achieve localization accuracies comparable to individual MRI-based solutions in many cases. This has important implications for research settings where individual MRIs are unavailable or impractical.

The manuscript unfolds with a concise literature review regarding EEG source localization in Section 2, followed by Sections 3, 4, elucidating the methods that form the core of the aggregated pipeline and its evaluation. The method's section leads to the presentation of results in Section 5, followed by a discussion in Section 6 presenting the contributions to the body of knowledge, and open works for the future in Section 7. The SL pipeline is available as a Python package (Github).

## 2 Related work

Neuro-imaging uses various techniques to directly or indirectly capture the nervous system's structure and function. The origins of neuro-imaging can be traced back to the 1880s, with the pioneering work of Angelo Mosso, an Italian physiologist (Mosso, 1884). He developed the “human circulation balance,” a non-invasive way of measuring blood flow to the brain (Sandrone et al., 2012). Following Mosso's innovation, various other techniques for exploring the live human brain emerged. These included the use of X-rays toward the end of the 19th century (Morton, 1896; Assmus, 1995), the introduction of air ventriculography in 1918 (Dandy, 1918), and the development of electroencephalography (EEG) in the 1920s by German psychiatrist Hans Berger (Berger, 1930; Tudor et al., 2005). EEG is a fundamental technique for exploring the inner workings of the human brain. This non-invasive technique captures electrical signals generated by neuronal activity, providing insights into the brain's inner complex workings with remarkable temporal resolution and a relatively low cost. EEG's effectiveness, versatility and safety have cemented its place in research and diagnostic settings, making it an invaluable tool for understanding brain function and diagnosing neurological disorders (Adeli and Ghosh-Dastidar, 2010; Alturki et al., 2020). However, EEG's effectiveness is hampered by its limited spatial resolution, which presents challenges in applications that demand accurate identification of neural sources.

Source localization enhances EEG's spatial resolution, bridging the gap between its excellent temporal resolution and its traditionally limited spatial accuracy. By determining where the brain-specific electrical activities originate, researchers and clinicians can gain deeper insights into the neural underpinnings of cognitive processes, behaviors, and disorders such as Alzheimer's (Aghajani et al., 2013), depression (Zhu et al., 2018), or focal epilepsy (Plummer et al., 2008) to name a few. Given the nature of this problem, it is unfeasible to precisely locate the source of brain activity from scalp-level sensor data (Vogel, 2002; Aster et al., 2018), given there are infinitely many possible electrical source configurations that could have given rise to the recorded potentials at the electrode level. While pinpointing exact activation sources is not feasible, approximations can be made by making assumptions and imposing constraints. EEG source localization methods seek to pinpoint the origins of electrical activity within the brain. Various source localization (SL) methods have been developed since the 1970s to overcome this issue.

The first successful attempt to create such a heuristic and produce quantitative findings was documented by Schneider and Gerin (1970). The initial strategy for SL involved modeling the human head as a uniformly conductive sphere with a single electrical dipole inside and electrodes on its surface. Solving the forward problem—calculating current flow from the dipole to the electrodes—is relatively simple using electrical theory (Smythe, 1988). However, solving the inverse problem—tracing currents from the electrodes back to their sources—is complex due to the countless dipole configurations that could lead to the observed scalp potentials. A unique solution becomes attainable only when the problem is confined to a parametric model (Schneider, 1972). EEG SL algorithms require a source equation (forward model) to compute the potential at any possible electrode site and the equations for the partial derivatives of each parameter. Iteratively adjusting model parameters, like the dipole moment, and comparing the model's output with actual scalp potentials, the model's accuracy can be progressively enhanced until, eventually, the algorithm converges and a solution is found (Marquardt, 1963). Another early approach tried using an inhomogeneous sphere model with different conductivity layers (Rush and Driscoll, 1969). Although this model yielded slightly better results, its increased complexity led to poorer computational performance. Consequently, simpler homogeneous models were favored, although they do not accurately represent the inhomogeneity of actual human heads (Kavanagk et al., 1978).

The aforementioned family of SL methods, known as dipole-based, attempts to model brain electrical fields by locating one or a few dipoles inside the subject's head. Dipole-based approaches have proven to be good approximations of brain electrical current generation and have been extensively used in literature (Cuffin, 1998). In 1984, a new family of SL methods was introduced, known as distributed or non-parametric methods. The shift from a parametric (few-dipole) to a non-parametric (distributed sources) approach represents a significant change in how the inverse problem is addressed. In a parametric approach, the challenge is to estimate a small number of parameters, such as the location, orientation, and magnitude of a few dipoles, based on the observed data. This method is constrained by the need to correctly specify the number and approximate location of these dipoles a priori, which can be highly challenging and subject to error. In contrast, distributed source models do not pre-specify the number and exact locations of the sources. Instead, they treat the entire brain or a large area of interest as a grid of potential sources (thousands of dipoles) and estimate the activity level at each point. This paradigm shift from dipole-based to distributed-based approaches was mainly due to advances in mathematical and computational methods such as singular value decomposition (Stewart, 1993). Distributed approaches inherently provide a more comprehensive and detailed brain activity mapping. Still, it introduces a high degree of ill-posedness to the inverse problem, as the number of unknowns (sources) significantly exceeds the number of observations (electrodes).

Minimum Norm Estimates (MNE) (Hämäläinen and Ilmoniemi, 1994) emerged as the pioneering distributed method for modeling brain activity, marking a significant departure from the limited dipole-based approaches of earlier techniques by proposing that the sources of measured electromagnetic fields are spread across numerous potential locations within the brain. Despite its innovation, MNE is known for its propensity to misplace deep brain sources, inaccurately attributing them to superficial cortical areas, a drawback extensively documented in subsequent analyses (RD, 1995; Pascual-Marqui, 1999). The LORETA algorithm (Pascual-Marqui et al., 1994) was introduced in 1994 to address this limitation, improving deep source localization. Further advancements led to the development of standardized LORETA (sLORETA) (Pascual-Marqui et al., 2002) and exact LORETA (eLORETA) (Pascual-Marqui, 2007), each refining the approach to source localization with enhanced accuracy and specificity, thereby overcoming some of the fundamental constraints observed in the original MNE methodology.

The validation of source localization methods presents significant challenges due to the absence of direct ground truth in non-invasive recordings. Previous validation approaches typically fall into four categories, each with limitations for naturalistic applications.

Approaches for developing and testing source localization algorithms span averaged trials based on events (Slotnick, 2004; Tsolaki et al., 2015), integration with fMRI and other neuro-imaging modalities (Ritter and Villringer, 2006; Huster et al., 2012), simulations (Supek and Aine, 1993; Yao and Dewald, 2005; Dümpelmann et al., 2009), and intracranial EEG recordings (Bénar et al., 2006; Plummer et al., 2008; Michel and Brunet, 2019).

Simulation studies (Supek and Aine, 1993; Yao and Dewald, 2005; Dümpelmann et al., 2009) provide controlled environments with known ground truth but may inadequately represent real EEG characteristics. Event-Related Potential (ERP) paradigms (Slotnick, 2004; Tsolaki et al., 2015) analyze brief EEG windows relative to specific events, reducing noise through trial averaging but potentially limiting generalizability to single-trial continuous EEG. Multi-modal validation approaches incorporate complementary neuroimaging methods, particularly fMRI (Bénar et al., 2006; Grova et al., 2008; Bak et al., 2011). Work by Hasson et al. (2004) introduced Inter-Subject Correlation (ISC) for analyzing congruence in brain activations during naturalistic viewing, finding substantial correlation among viewers watching identical film segments. This approach revealed altered cortical activity patterns in autism (Hasson et al., 2009) and depression (Gruskin et al., 2020). However, Hasson et al. (2010) noted limitations in identifying common activation patterns during naturalistic viewing, particularly the challenge of controlling specific brain areas due to stimulus complexity. Finally, invasive validation through stereotactic EEG (Bénar et al., 2006; Mikulan et al., 2020) offers direct measurement but involves surgical electrode implantation and may generate electrical impulses differently than natural neural activity.

A promising alternative validation approach focuses on neurophysiological plausibility—whether source localization results align with established knowledge of functional neuroanatomy during specific tasks. Several researchers have employed this approach, particularly for visual processing tasks. Cottereau et al. (2012) demonstrated that source-localized EEG could accurately map retinotopic organization in visual cortex, corresponding to known visual processing architecture. Brodbeck et al. (2018) showed that temporal responses in localized auditory regions during speech processing matched expected processing hierarchies.

More relevant to naturalistic paradigms, Vanderwal et al. (2019) demonstrated that video viewing produces reliable activations in visual processing regions using fMRI. These findings suggest that similar neurophysiologically valid activation patterns should be detectable using source-localized EEG if methods are robust. Critically, Michel et al. (2004) argued that the neurophysiological plausibility of source localization results can serve as a meaningful validation metric when direct ground truth is unavailable.

Whether individual MRIs are necessary for accurate source localization remains unclear. Valdés-Hernández et al. (2009) found that template-based approaches could achieve localization accuracies comparable to individual MRI-based solutions in many cases. Song et al. (2015) demonstrated that standardized head models can provide reliable source estimates when individual MRI data is unavailable, though with some reduction in precision. Akalin Acar and Makeig (2013) systematically evaluated the impact of using template versus individual MRIs on source localization accuracy, finding that while individual MRIs improved results, template-based approaches still produced neurophysiologically plausible activations for well-understood tasks.

Despite these advances, a critical gap remains in validating source localization using unimodal EEG without subject-specific anatomical information in naturalistic settings. While controlled laboratory studies have demonstrated the theoretical accuracy of various methods, the performance of source localization with continuous, non-event-locked data in ecological settings remains underexplored. Previous work has typically relied on averaged responses, multi-modal integration, or highly controlled stimuli, limiting generalizability to real-world applications.

Our work addresses this gap by evaluating whether established source localization methods, when applied to naturalistic EEG data without subject-specific information, can produce activation patterns that align with neurophysiological expectations. Rather than developing novel algorithms, this research work focuses on assessing whether existing methods can generate plausible results under conditions that more closely approximate real-world research settings, using task-induced neural differences as a validation proxy.

## 3 Aggregated pipeline design

The aggregated pipeline consists of two key components: The automatic EEG-preprocessing strategy. This section provides an overview of the pre-processing and source localization methods. Further design details are provided as Supplementary Section 1.

### 3.1 EEG preprocessing

Pre-processing of EEG signals is a crucial step for any subsequent analysis. In the case of EEG source localization, it is particularly relevant given that artifact-free recordings in the sensor space lead to a cleaner signal in the source space. A general, automatic pre-processing strategy should preserve the cognitive information in the raw EEG signals while removing the artifacts caused by internal and external sources such as eyeblinks or electrode displacement. The current study implements a general-purpose pre-processing strategy based on established guidelines (Miyakoshi et al., 2007; Makoto, 2024).

**Figure 1:**
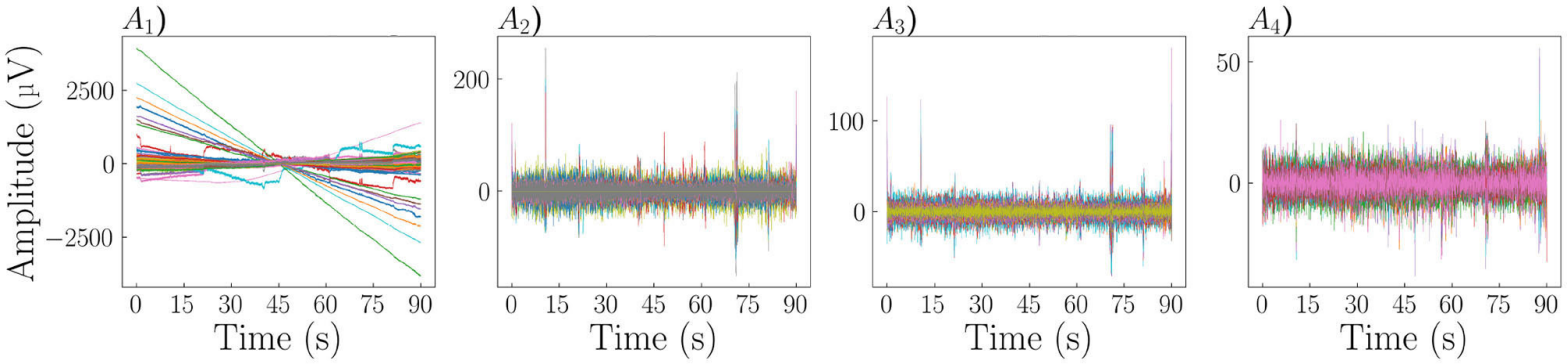
Pre-processing steps. **(A1)** Downsample to 125Hz (DC offset removed for plotting). **(A2)** Band pass filter (1,50 Hz). **(A3)** PREP pipeline for bad channel detection and interpolation, line noise removal, and robust average re-reference. **(A4)** Automatic ICA-based artifact removal.

This strategy, depicted in Figure 1, can be summarized as follows: (Figure A1) Downsampling EEG data; (Figure A2) Bandpass filtering; (Figure A3) Bad channel detection and interpolation; and (Figure A4) Artifact correction. The detailed description of pre-processing methods as well as their validation is provided as Supplementary Section 1.1

### 3.2 EEG source localization

EEG source localization aims to estimate the location and strength of neural sources within the brain that give rise to the observed scalp potentials. This process addresses two main problems: a *forward model* to determine how current travels from the sources to the scalp and an *inverse method* that estimates source activation given scalp-level recordings.

#### 3.2.1 B1 - Forward model

**Figure 2:**
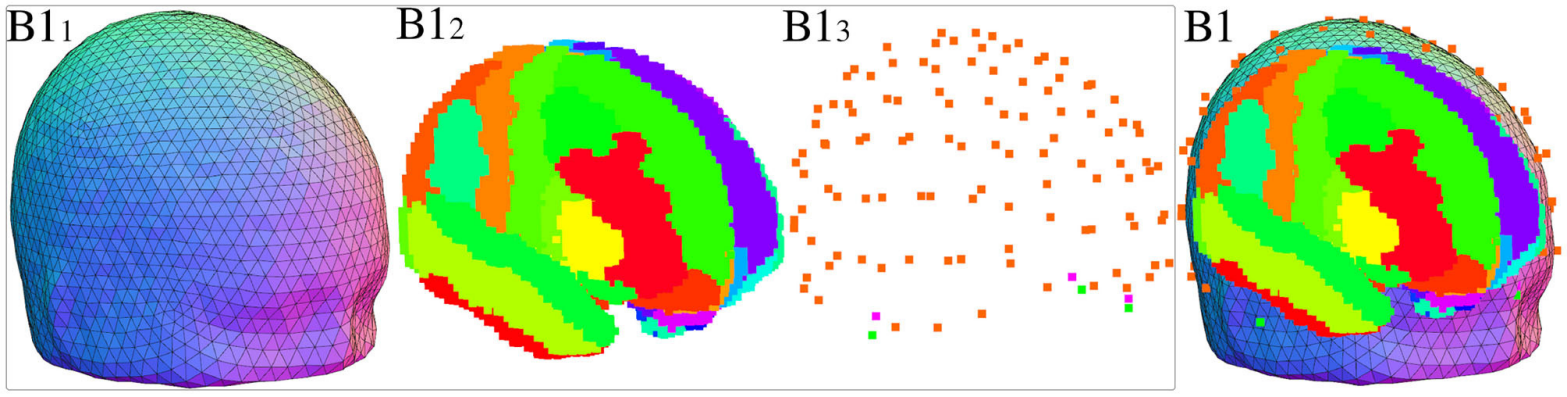
**(B1)** Forward model, made up of **(B1**_**1**_**)** Boundary Element Model (BEM), **(B1**_**2**_**)** source space, and **(B1**_**3**_**)** electrode montage.

A forward model (Figure 2B1) is derived from the average ICBM 2009c Nonlinear Symmetric template (Fonov et al., 2009) in conjunction with the CerebrA atlas (Manera et al., 2020) and standard electrode positions and applies inverse modeling through the eLORETA algorithm. The forward model is made up by the Boundary Element Model (Figure 2B1_1_), Source space (Figure 2B1_2_), and electrode montage (Figure 2B1_3_).

The forward model can be computed using the freely available MNE-Python software package (Gramfort et al., 2013), which implements efficient algorithms for calculating the lead field based on the Boundary Element Method (BEM), the defined source space, and electrode locations. The resulting forward model allows for estimating EEG signals for a given source space distribution, forming the basis for the inverse problem solution.

#### 3.2.2 B2 - Inverse method

The exact low-resolution electromagnetic tomography (eLORETA) method is chosen for the inverse solution. This decision is based on several factors. The established eLORETA algorithm has been extensively validated through simulations, controlled experiments, and multi-modal approaches. eLORETA has been shown to provide unbiased localization even in the presence of structured noise and multiple sources, and its properties make it well-suited for analyzing continuous EEG data not based on trials (Pascual-Marqui, 2007). While eLORETA is the primary choice, the proposed aggregated pipeline is designed to be flexible, potentially allowing for other inverse methods if required for specific applications or comparative studies.

In the context of eLORETA, the parameter λ^2^ plays a critical role in balancing the trade-off between data fidelity and the regularization term, which accounts for noise and model complexity. The choice of $\lambda^{2}=\frac{1.0}{{\text{SNR}}^{2}}$ is a judicious design decision, derived from the signal-to-noise ratio (SNR):


(1) $$\begin{array}{l} \text{SNR}=10\cdot \log_{10}\left(\frac{\bar{P}}{\sigma^{2}}\right) \end{array}$$

Where $\bar{P}$ is the mean power of the EEG signal and σ^2^ is the variance of the signal power. By defining λ^2^ as the inverse square of the SNR, the parameter effectively adapts to the quality of the signal: higher SNR values, indicating cleaner signals, result in smaller λ^2^ values, thus reducing the regularization effect and allowing the algorithm to rely more on the observed data. Conversely, lower SNR values, indicative of noisier signals, lead to larger λ^2^ values, thereby increasing the regularization term and preventing overfitting to the noisy data.

## 4 Instantiation and empirical design

**Figure 3:**
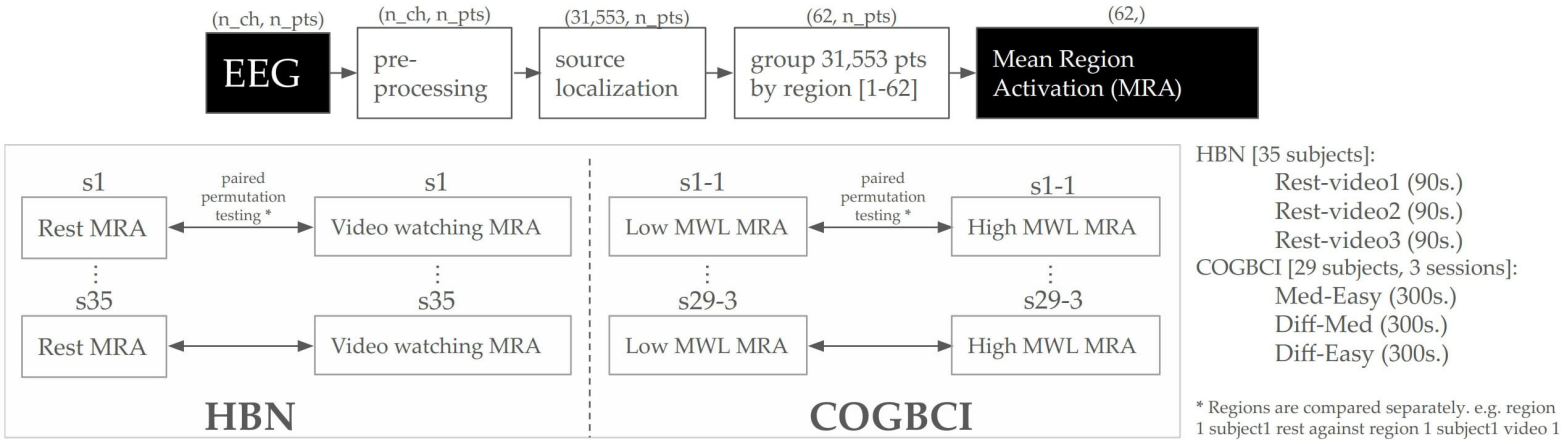
Overall evaluation design. EEG signals are pre-processed, localized, and grouped into 62 unequally sized cortical regions. The mean amplitude value for each cortical region across time is computed (MRA). Paired permutation testing is used to test for task-related significant differences in region activation.

This section describes the methods used to assess the performance and effectiveness of the proposed aggregated pipeline for EEG source localization. Because EEG recordings lack an unequivocal ground-truth source space, direct validation of how closely the reconstructed sources match actual neural activations is not possible. Instead, this study compares source space activations across different tasks, leveraging task-induced variability as a proxy measure. Specifically, if the proposed method is effective, it should retain neural patterns associated with distinct tasks, even without subject-specific anatomical data. Figure 3 depicts the evaluation methodology. EEG signals are pre-processed, localized, and grouped into cortical regions. The mean activation within each cortical region is computed and permutation testing is employed to test for statistically significant task-related differences in region activation.

### 4.1 Data acquisition

The HBN (Healthy Brain Network) and COG-BCI datasets are employed to evaluate the aggregated pipeline. Both feature high-density electrode montages, as higher electrode counts are known to improve source localization accuracy (Lantz et al., 2003). The forward model generation for each distinct dataset is based on their expected electrode locations. Details on how the montages are defined and aligned to the head model are provided as Supplementary Section 1.2.1.

#### 4.1.1 HBN dataset

The Healthy Brain Network (HBN) biobank represents one of the most extensive open-access pediatric psychiatric datasets, comprising multi-modal data from 10,000 participants (ages 5–22) in the New York City area (Alexander et al., 2017). While the biobank includes neuroimaging, genetic, and phenotypic data, this analysis focuses on the high-density EEG recordings collected using a 128-channel EGI system with a sampling rate of 500 Hz. This biobank offers multi-modal data such as fMRI recordings and genetic and phenotypical information, but only EEG data was used.

One limitation of the proposed aggregated pipeline is that it applies only to adults. The average brain template (MNI-ICBM152-2009c) was computed only with adult MRIs. Consequently, thirty-five young adults (ages 18–22) who completed resting-state and naturalistic viewing paradigms were selected. The resting-state recordings consist of 90-second epochs with participants maintaining a relaxed state with eyes open. The naturalistic viewing paradigms include three distinct video stimuli: a movie trailer (“Diary of a Wimpy Kid,” 115 seconds), an educational clip (“Fun with Fractals,” 150 seconds), and a feature film excerpt (“Despicable Me,” 150 seconds). To facilitate direct comparisons across conditions, all video-related EEG recordings were truncated to match the 90-second duration of the resting-state data. This dataset is particularly valuable due to its combination of resting and engagement-driven conditions, which provides a basis for assessing how well the method handles task-free and naturalistic EEG data.

#### 4.1.2 COGBCI dataset

The COG-BCI database is the second validation dataset, offering controlled cognitive paradigms designed to elicit distinct mental states. This dataset comprises EEG recordings from 29 participants (11 female, 18 male, mean age 23.9 ± 3.20 years) across three separate weekly sessions, using a 64-channel ActiCap system with active Ag-AgCl electrodes and an ActiCHamp amplifier sampling at 500 Hz. While the dataset includes multiple experimental paradigms, the validation focuses on two key components that provide complementary insights into the methods' effectiveness: the MATB-II task and the resting state recordings.

The MATB-II (Multi-Attribute Task Battery) offers a more ecologically valid scenario, requiring participants to simultaneously manage four aviation-related subtasks: system monitoring, tracking, communications, and resource management. Three distinct difficulty levels (easy, medium, and difficult) were implemented through systematic task combinations, with each 5-min block generating increasingly complex EEG patterns and movement-related artifacts. This multi-tasking paradigm provides an ideal test case for pre-processing robustness under conditions more closely approximating real-world cognitive demands.

Additionally, the dataset includes standardized resting state recordings consisting of one-minute eyes-open and one-minute eyes-closed conditions at the beginning and end of each session. These baseline measurements, collected under controlled conditions identical to those in the HBN dataset, enable direct cross-dataset comparisons and validation of pre-processing stability across different experimental contexts.

A comprehensive validation framework was created by combining the naturalistic data from HBN with the controlled paradigms from COG-BCI. This dual approach tests the pipeline under ecologically valid task conditions and more experimentally constrained scenarios.

### 4.2 Evaluation

The aggregated pipeline evaluation comprises two principal components: EEG pre-processing assessment and cross-task analysis through permutation testing. The EEG pre-processing assessment is provided as Supplementary Section 2.2.

Permutation testing represents a robust non-parametric statistical methodology suited for electroencephalographic (EEG) data analysis (Maris and Oostenveld, 2007). This approach offers significant advantages due to its resilience against violations of normality assumptions and its inherent capacity to address multiple comparisons. The methodology's core principle involves randomizing condition labels to generate a null distribution, which subsequently serves as a reference for evaluating observed between-condition differences. The method's suitability for EEG analysis stems from its distribution-free nature and ability to account for neurophysiological signals' complex temporal and spatial correlations. In this implementation, 10,000 iterations were selected as an optimal balance between computational efficiency and statistical precision. This quantity enables the detection of significance levels reaching 0.0001 (1/10, 000) while ensuring stable p-value estimations. While p-value estimation accuracy correlates positively with the number of permutations, empirical evidence suggests that exceeding 10,000 iterations yields diminishing returns in neuroscientific applications.

The permutation testing procedure employs a paired design, wherein label shuffling occurs within subjects at each iteration. For instance, S1-Rest may be permuted with S1-Video1 but never with S2-Rest or S2-Video1. The difference between means serves as the test statistic, with the empirical H0 assuming no difference in the source activation space means. Two thresholds, set at the 2.5th and 97.5th percentiles of H0, establish significance, equivalent to an alpha value of α = 0.05 in a two-tailed normal distribution.

The inverse model application at each time step generates a source activation space containing 31, 553 source activation points. This produces a *source space activation matrix* **S** ∈ ℝ^*i, t*^, where *i* represents an activation point in space and *t* denotes a time step. For the HBN dataset's resting conditions, lasting 90 seconds and downsampled to 125Hz, this results in 11,250 time steps, yielding a matrix of 31, 553 × 11, 250 real numbers representing nano ampere per meter (nA/m), the output of the eLORETA method.

The evaluation procedure examines source space differences through two approaches. First, it computes the mean of the source space for a particular sample, resulting in a single scalar for task difference comparison. Second, it groups source space by region [0–61], computing the mean of each region across time to produce a 62-dimensional vector. The 31,553 sources are grouped into 62 differently-sized cortical areas, with regional means computed across all time points within each condition.

For the HBN dataset, permutation testing examines the following condition pairs: Rest vs. Video1, Rest vs. Video2, and Rest vs. Video3. The COGBCI dataset analysis compares Medium vs. Easy, Difficult vs. Medium, and Difficult vs. Easy conditions.

### 4.3 Hypotheses

Based on the methodological framework and the datasets employed, two main hypotheses were formulated to validate the neurophysiological plausibility of the source localization results:

The first hypothesis focuses on the pipeline's ability to distinguish between task-based and resting-state conditions in the HBN dataset. Previous analyses of this dataset at the sensor level have demonstrated apparent differences between video-watching and resting-state conditions (Alexander et al., 2017). Given these established sensor-level distinctions, these differences are expected to be preserved and potentially more precisely localized in source space, aligning with an established neurophysiological understanding of visual processing pathways.

The position of each region within the cortex is included as Supplementary Figure S5, with 62 cortical regions as defined in the CerebrA atlas (Manera et al., 2020). In this specific experiment, the cortical regions associated with visual processing and attention during the video-watching state are particularly interesting in this analysis. The posterior region of the brain, particularly the occipital lobe, is known to be heavily involved in visual processing (Grill-Spector, 2003). Therefore, while watching a video, increased activation in this area is expected due to the visual nature of the condition (Hasson et al., 2004). Additionally, the parietal lobe, located in the posterior region, plays a crucial role in attention and spatial processing (Corbetta and Shulman, 2002), is likely to be activated in this condition. Furthermore, fMRI studies during naturalistic video watching have consistently shown increased activation in posterior brain regions (Rees, 2007). These findings collectively support the informal hypothesis that the posterior part of the brain is more activated in the task condition than in the resting condition. In other words, a significant positive correlation between the difference in rest-task conditions for particular regions and their positions on the Anterior-Posterior (Y) axis is expected to be observed.

**H_1_**: Source reconstructions from video-watching tasks will show significantly higher activation than resting-state conditions, with particularly pronounced differences in posterior brain regions associated with visual processing.

The second hypothesis examines the pipeline's sensitivity to varying cognitive workload levels using the COGBCI dataset. Prior analysis of this dataset has demonstrated reliable sensor-level differences between workload conditions. These differences align with established theories of cognitive load and attentional networks. By extending these findings to source space, the aggregated pipeline's ability to capture functionally relevant neural activity patterns can be validated.

**H_2_**: Source reconstructions will show progressive increases in activation intensity corresponding to increasing cognitive workload levels, with the highest workload condition showing significantly greater activation compared to both medium and low workload conditions.

These hypotheses collectively address two validation aspects. (1) differentiating between task-specific and resting-state neural activity patterns and (2) detecting gradual changes in brain activation corresponding to varying cognitive demands. These capabilities are essential for establishing the pipeline's utility in practical neuroscience applications and its potential for advancing our understanding of brain dynamics during different cognitive states.

### 4.4 Software and hardware

The software implementation utilized Python 3.10 as the primary programming language, with MNE 1.6.0 as the core EEG data processing framework. The software stack included essential scientific computing libraries such as Pandas for data manipulation, NumPy for numerical operations, and Matplotlib for visualization. Three-dimensional brain visualizations were created using Open3D, while Freesurfer was employed for deriving the necessary geometries from structural MRIs.

The computational infrastructure comprised a workstation with an Intel i9-12900k processor and 64GB of RAM. Source localization processing time averaged approximately two minutes per five-minute sample. The data management requirements were substantial, with the total data volume reaching several terabytes across all experiments.

The memory requirements for data processing were significant due to the high-dimensional nature of both sensor-level and source-space data. Each EEG sample at the sensor level, comprising 62 channels sampled at 125 Hz, required approximately 124 MB of storage. When transformed to source space with 31,554 points, the storage requirement increased to approximately 7.8 GB per sample. The total storage requirements amounted to 2.784TB for the COGBCI dataset, 800 GB for the HBN dataset, necessitating careful data management strategies and robust computational resources.

## 5 Results

This section presents the findings from evaluating the aggregated pipeline applied to the HBN and COGBCI datasets. The results are structured to provide an overview of the source space activation, the statistical validation of differences across conditions, and regional activation patterns. Statistical significance was assessed through permutation testing, and spatial correlations were evaluated to validate our hypotheses regarding task-specific activation patterns.

### 5.1 HBN dataset

The analysis of the HBN dataset aims to assess the pipeline's capacity to differentiate between resting-state and task-based neural activation patterns. This section presents the overall source space findings, statistical validation through permutation testing, and regional analysis of activation differences.

#### 5.1.1 Overall source space analysis

**Figure 4:**
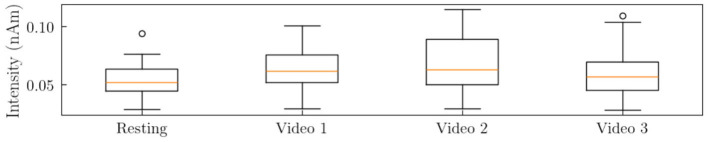
Boxplots depicting the distribution of the mean source space activation values across 35 subjects for each condition in the HBN dataset.

The global analysis of source space activation indicated a significant variation across the different experimental conditions. Figure 4 presents boxplots illustrating the distribution of source space activation means across 35 subjects for the resting and video-watching conditions.

The mean source activation values (mean ± standard deviation) for each condition were as follows: Rest (0.054 ± 0.013), Video1 (0.062 ± 0.016), Video2 (0.068 ± 0.022), and Video3 (0.059 ± 0.019). Initial inspection of Figure 4 reveals that the resting condition exhibited the lowest overall activation intensity in terms of mean and variance. The most pronounced difference in activation was observed between the Rest and Video2 conditions, whereas the most negligible difference occurred between the Rest and Video3 conditions.

**Figure 5:**
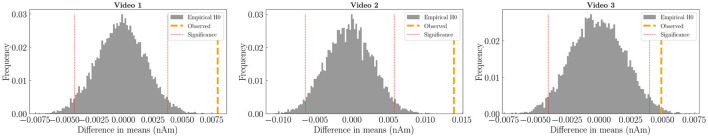
Permutation test across pairs of conditions. A significant difference is found in the means for all tested pairs of conditions (Rest-Video1, Rest-Video2, and Rest-Video3), since the observed mean for each video is above the 97.5th percentile.

Although visual inspection of the boxplots suggests overlapping distributions, formal statistical analysis using permutation testing demonstrated significant differences across conditions. Figure 5 presents the permutation test results, confirming the robustness of the observed effects.

As depicted in Figure 5, permutation tests demonstrated significant differences between all pairs of conditions (Rest-Video1, Rest-Video2, and Rest-Video3). The observed mean differences were 0.0079 nA/m, 0.0139 nA/m, and 0.0049 nA/m for Rest-Video1, Rest-Video2, and Rest-Video3 comparisons, respectively. In all cases, the observed differences (indicated by orange lines) exceeded the 97.5th percentile threshold (red lines), confirming significantly higher activation during video-watching compared to rest.

The within-subject analysis in the Supplementary Section 2.1 corroborates this trend. Most participants exhibited significantly higher activation in task conditions than in the resting condition. However, a few subjects displayed comparable or slightly elevated resting-state activation, highlighting inter-individual variability in neural response patterns.

#### 5.1.2 Per-region analysis

**Figure 6:**
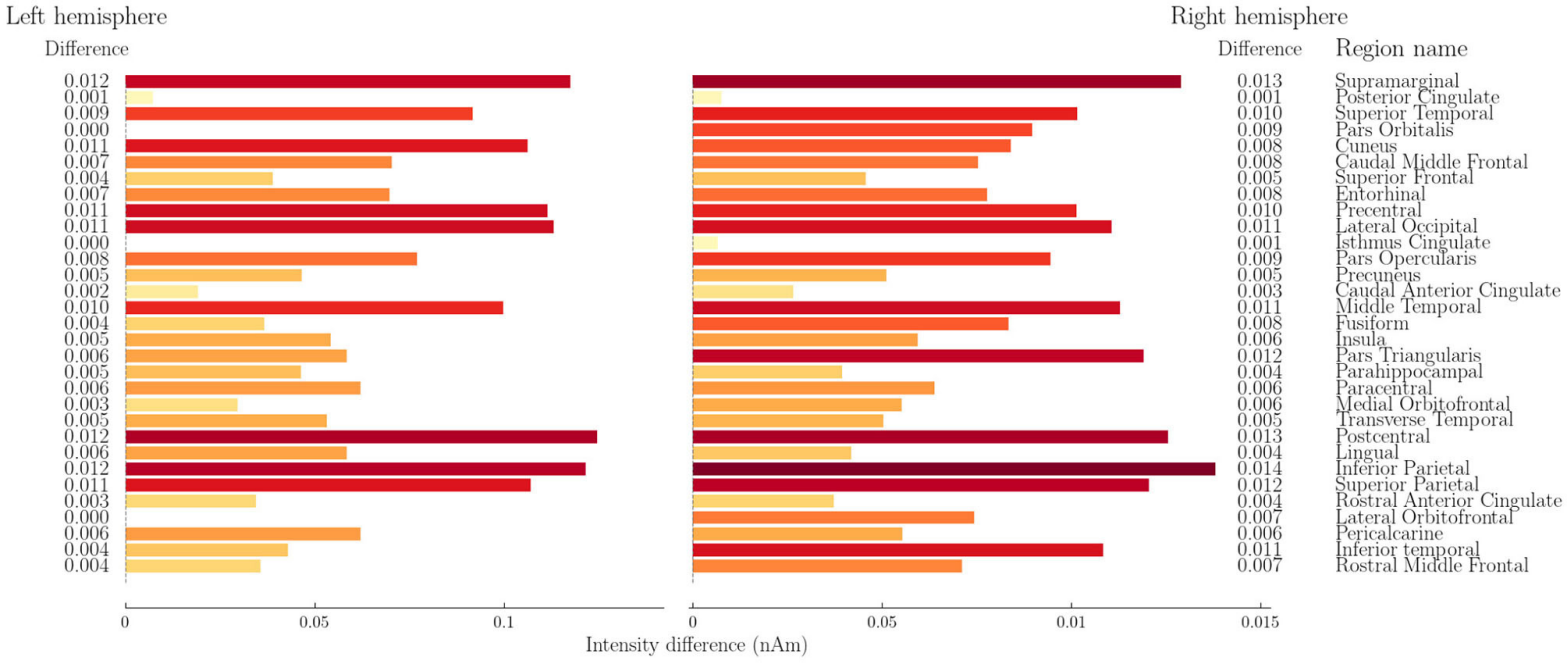
Difference (nA/m) between the source space intensity in resting condition vs video condition. Average of 3 videos. Non-significant differences are set to 0.

A detailed regional analysis was conducted to determine whether specific cortical areas exhibited significantly greater activation during task conditions compared to the resting state. The permutation test results provided confidence values indicating significant differences in regional activation. To facilitate interpretation, the results for the three video conditions were aggregated, with non-significant differences set to zero before averaging. Figure 6 presents the aggregated regional differences in source space activation.

**Figure 7:**
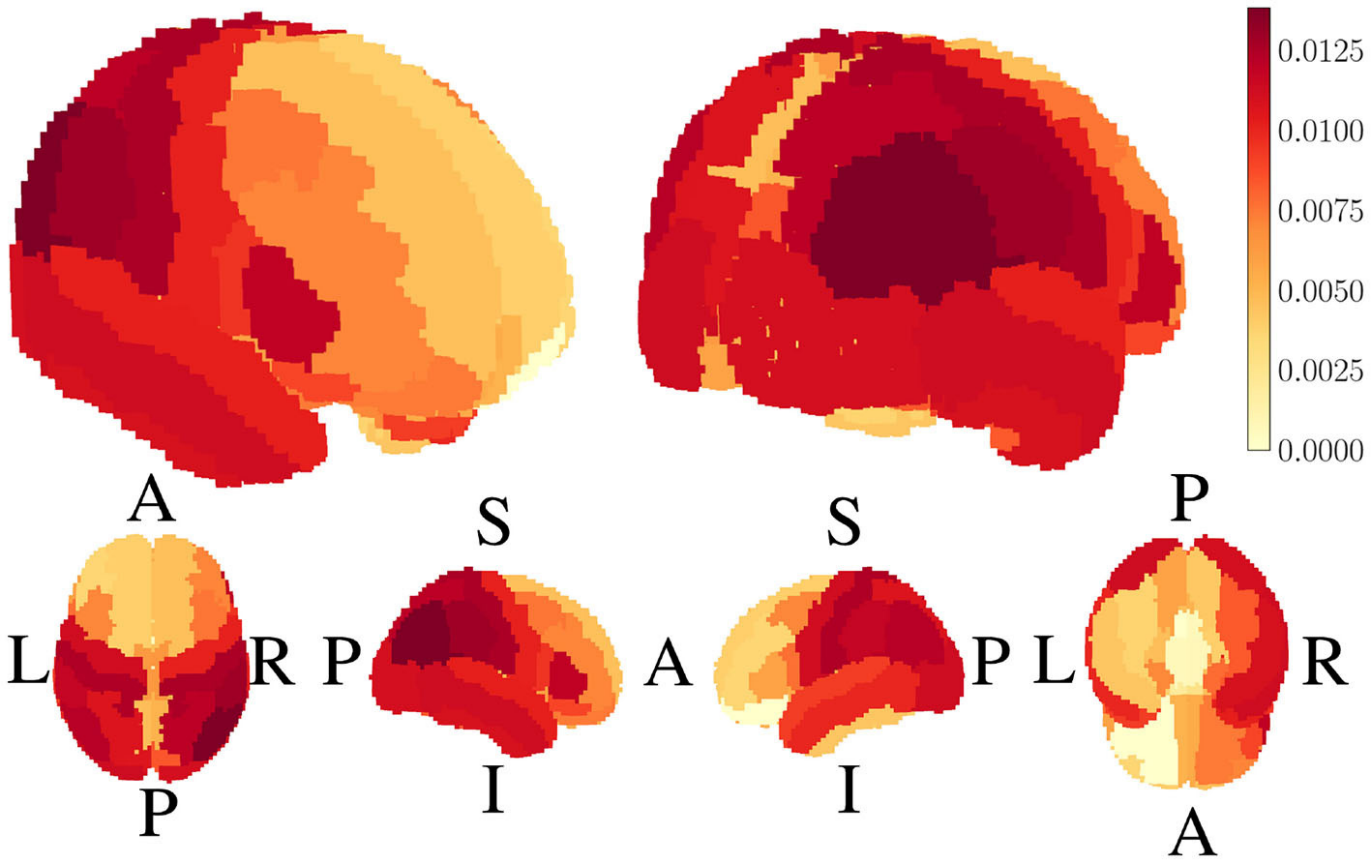
Spatial representation of the difference in activation intensity between resting and task conditions. Greater activation is observed in posterior regions, consistent with the involvement of visual processing areas (A, Anterior; P, Posterior; L, Left; R, Right; S, Superior; I, Inferior).

The most substantial activation differences were observed in the Supramarginal, Superior Temporal, Precentral, Lateral Occipital, Postcentral, and Inferior Parietal regions. Notably, a spatial visualization of these differences (Figure 7) highlights that the most pronounced increases in activation occur in the posterior regions of the brain, aligning with known visual processing pathways.

These findings are consistent with established neuroanatomical knowledge about brain function during visual processing tasks. The preferential engagement of posterior brain regions during video-watching tasks corresponds with the known roles of these areas in visual perception, attention, and sensory integration. The Lateral Occipital cortex, identified as one of the regions with substantial task-related activation increases, is a key component of the visual processing stream responsible for object recognition and visual feature integration. Similarly, the significant activation in the Supramarginal and Inferior Parietal regions aligns with their established role in visual attention and integrating visual information with other sensory modalities.

Statistical correlation analyses examined potential relationships between activation differences and regional characteristics. A Spearman correlation analysis revealed a moderate positive correlation between the activation difference and cortical region size (*r*_*s*_ = 0.479, *p* < 0.001), suggesting that larger cortical regions exhibit more significant differences in activation. Additionally, a weak-to-moderate positive correlation was observed between activation differences and a region's y-axis position (*r*_*s*_ = 0.323, *p* = 0.010). This further supports the hypothesis that posterior cortical regions preferentially engage in video-watching task conditions, which is neurophysiologically plausible given the visual nature of the stimuli.

#### 5.1.3 COGBCI dataset

The COGBCI dataset was analyzed to assess the pipeline's sensitivity to variations in cognitive workload. The results demonstrate a systematic increase in source space activation with increasing task difficulty, a neurophysiologically plausible pattern that aligns with known neural correlates of cognitive effort.

#### 5.1.4 Overall source space analysis

**Figure 8:**
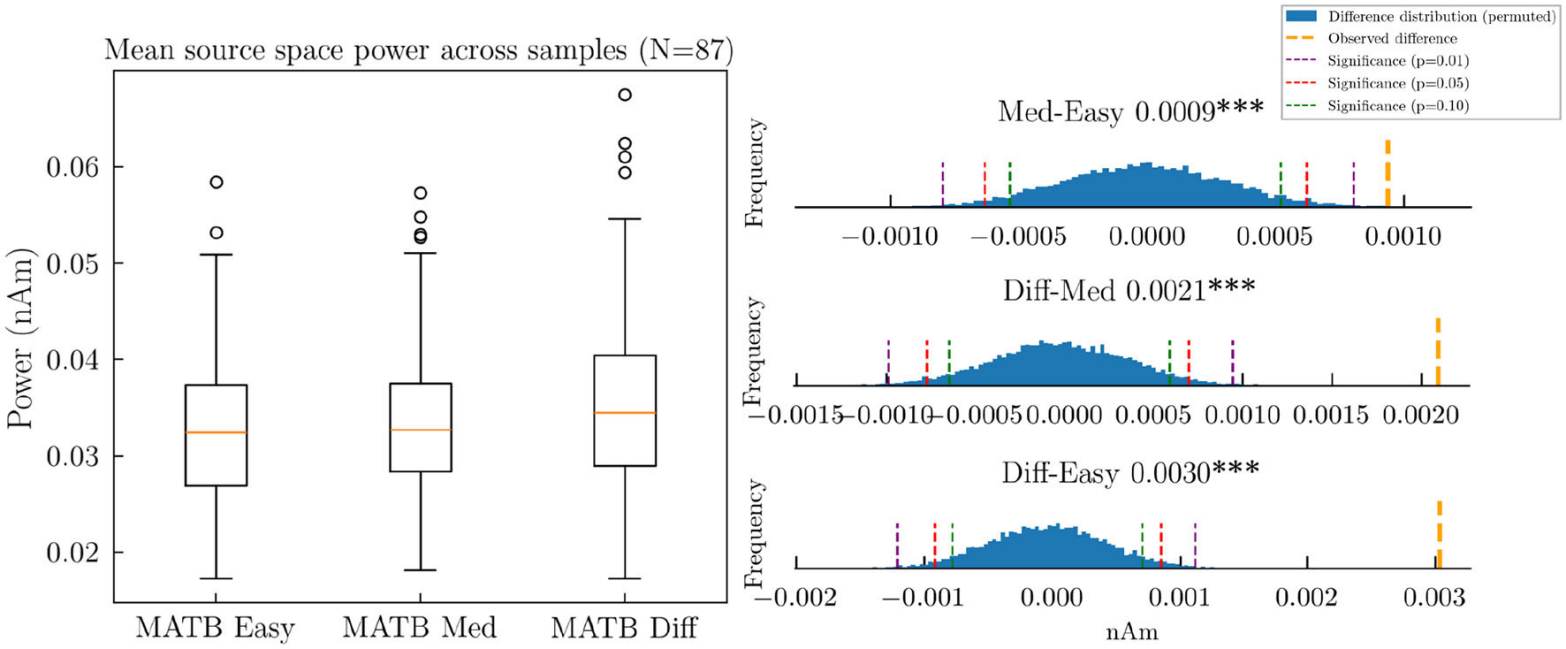
Mean source activation intensity (nA/m) across the three difficulty levels of the MATB task, demonstrating a progressive increase in mean source space activation with task difficulty. The triple asterisk (***) indicates a *p*-value smaller than 0.01.

Permutation testing revealed highly significant differences across all task conditions (*p* < 0.01). The mean source activation values (nA/m) were as follows: Easy (0.0328), Medium (0.0337), and Hard (0.0358). These findings indicate a progressive increase in cortical activation with increasing cognitive demand (Figure 8).

This graded response to cognitive load aligns with established neurophysiological principles of mental effort. As task demands increase, the brain recruits additional neural resources to maintain performance, leading to higher overall activation. This pattern has been consistently observed in neuroimaging studies using various modalities, including fMRI and PET. The present results demonstrate that source-localized EEG can capture this fundamental neurophysiological phenomenon even without subject-specific anatomical information.

#### 5.1.5 Per-region analysis

**Figure 9:**
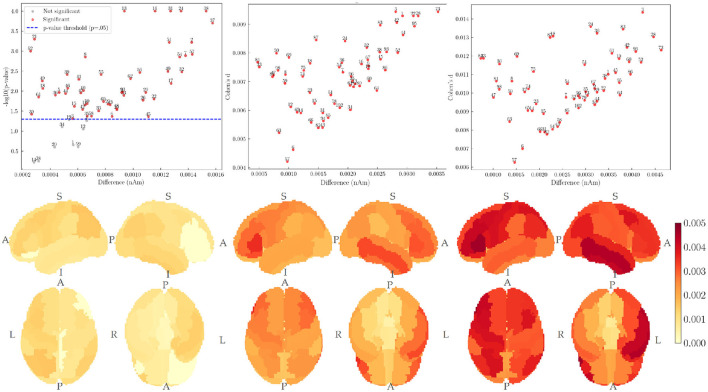
Difference (nA/m) between the source space intensity in MATB task conditions grouped by cerebral cortical regions.

A regional analysis further validated these findings, revealing increases in activation with task difficulty. These results align with established theories of cognitive workload and attentional control, emphasizing these cortical regions' role in executive function. Figure 9 presents a detailed visualization of regional activation differences across task conditions.

The systematic increase in activation with task difficulty was most evident in frontal and parietal regions involved in attentional control and working memory. This pattern provides strong neurophysiological support for the sensitivity of the aggregated pipeline to cognitive workload variations.

The least significant differences were found between the easy and medium conditions, with not all region differences being statistically significant (Figure 9 top-left). The comparison between medium and hard showed a statistically significant difference across all regions (Figure 9 top-mid, top-right).

The systematic progression of activation intensities across difficulty levels, combined with the anatomically specific distribution of these effects, strongly supports the hypothesis regarding the neurophysiological plausibility of the source-localized activity. The observed pattern—increasing activation with greater cognitive demand—represents a fundamental aspect of brain function that has been extensively documented in the cognitive neuroscience literature. The fact that this pattern emerges clearly from the analysis, despite the absence of subject-specific anatomical information, provides compelling evidence for the validity of the source localization approach in naturalistic settings.

Particularly noteworthy is the spatial distribution of these workload-related activation increases, which predominantly involve regions of the frontoparietal network known to be critical for cognitive control, attentional allocation, and working memory. This anatomically specific pattern further substantiates the neurophysiological plausibility of the results and demonstrates that source localization using standardized head models can capture meaningful functional neuroanatomy.

## 6 Discussion

This section aims to discuss the results presented in Section 5 and contextualize them within the existing body of knowledge. The study sought to evaluate whether established source localization methods could produce neurophysiologically plausible results without subject-specific anatomical data or manual intervention. The findings support the main hypotheses and demonstrate that these methods can effectively retain EEG information from the sensor space at the source space level while producing activation patterns consistent with known functional neuroanatomy.

A significant increase in the SNR was observed, as indicated by the dependent *T*-tests between consecutive pre-processing steps. The band-pass filter demonstrated the most significant effect on the SNR. This increase can be attributed to several factors, such as reducing the signal's slow drift and other low-frequency artifacts. The high pass filter is also believed to have increased SNR by removing high frequencies unrelated to cognitive processes, such as artifacts caused by muscle movements or electrical interferences. Visual inspection of the automatically removed components confirmed that this final step benefits the SNR. Overall, the employed pre-processing strategy has proven to increase the quality of the EEG signal, preserving cognitive information while reducing various artifacts. The approach does not require human intervention, making it suitable for long EEG recordings. The absence of artifacts and other forms of noise also enables a meaningful subsequent source localization and, therefore, a robust estimation of neural sources.

The first hypothesis, predicting differentiation between task-based and resting-state conditions in the HBN dataset, was strongly supported. The permutation tests revealed significant differences in source space activation between resting and video-watching conditions, with particularly pronounced effects in posterior brain regions associated with visual processing. The observed pattern of increased activation in occipital and parietal areas during video watching aligns well with an established understanding of the brain's visual processing hierarchy. The positive correlation between activation differences and posterior positioning of brain regions (*r*_*s*_ = 0.323, *p* = 0.010) provides further validation that standardized source localization approaches can detect functionally relevant patterns of neural activity without requiring individual anatomical data.

Significant differences in source space activation were observed for every region in the conditions. The difference was most prominent in the Supramarginal, Superior Temporal, Precentral, Lateral Occipital, Postcentral, and Inferior Parietal regions. Visual inspection of these activations showed a larger difference toward the posterior part of the head. These results align with expectations based on previous neuroimaging studies. The greater activation observed in posterior regions in the task condition (video watching) is consistent with the known involvement of these areas in visual processing and attention. The occipital lobe, located in the posterior part of the brain, is primarily responsible for visual processing. Additionally, the parietal lobe, situated posteriorly, plays a crucial role in spatial attention and integration of sensory information.

Intuitively, in these specific cases in which the activation in the resting condition was equal or higher than the activation of at least one condition might be explained by the notion of mind-wandering (Seli et al., 2016; Christoff et al., 2016). In other words, participants who were asked to relax in the resting condition might have entered the mind-wandering state, producing intentional or unintentional thoughts, endogenous (voluntary), and exogenous (involuntary), reflecting a higher activation of the source space. In particular, for half of the subjects, the activation in the task conditions was always significantly higher than in the resting condition (16 cases out of 35). For some subjects, the overall activation in the resting condition was larger than all the activation of the task conditions (4 cases). Additionally, for some subjects, the activation of the resting condition was greater than or equal to at least one task condition (9 cases) or equal to at least one (7 cases). Similarly, they might have entered such a state in the task conditions because these were not directly demanding a response, meaning the selected task did not require explicit responses from participants. Eventually, the analyzed EEG segments were 90 seconds long; therefore, in some cases, there might not have been enough data to demonstrate that the task conditions always lead to higher activation of the source space when compared to the resting condition.

The second hypothesis, regarding sensitivity to varying cognitive workload levels, was also supported by the COGBCI dataset results. The systematic increase in source space activation with task difficulty demonstrates the ability to detect graded neural activity changes. This finding is particularly noteworthy as it suggests that established source localization methods can capture subtle variations in cognitive state without requiring averaging across multiple trials or manual pre-processing. The progressive increase in activation with cognitive demand aligns with established neurophysiological models of attentional control and executive function, providing further evidence for the neurophysiological plausibility of the results.

A key strength of the employed approach is its ability to operate effectively with unimodal EEG data. While previous approaches often relied on multimodal imaging or extensive manual preprocessing, the results demonstrate that meaningful source localization can be achieved using standardized head models and automated preprocessing steps. The successful differentiation of experimental conditions across two independent datasets validates this approach.

The observed correlation between regional activation differences and cortical region size (*r*_*s*_ = 0.479, *p* < 0.001) warrants further investigation. While this relationship could reflect genuine properties of neural activation patterns, it might also indicate methodological considerations in how standardized approaches handle regions of different sizes. Future work should examine whether this relationship holds across different experimental paradigms and analysis approaches.

While individual anatomical variations can influence source localization accuracy, standardized head models remain viable for many research applications. The validation across two independent datasets demonstrates consistent and interpretable activation patterns, suggesting that template-based source localization can effectively capture meaningful neural activity patterns. This finding aligns with previous research by Valdés-Hernández et al. (2009), who showed that template-based approaches could achieve localization accuracies comparable to individual MRI-based solutions in many cases. Similarly, Song et al. (2015) demonstrated that standardized head models can provide reliable source estimates when individual MRI data is unavailable. The present approach builds upon these established findings while addressing practical constraints often encountered in research settings, such as limited access to MRI facilities, cost considerations, and time constraints. The robust performance observed across demographically diverse datasets supports the utility of this standardized approach, particularly in contexts where individual structural imaging is not feasible or practical.

The primary contribution of this study is demonstrating that established source localization methods, when applied without subject-specific information, can produce neurophysiologically plausible activation patterns that align with expected functional neuroanatomy. The observed posterior activation during visual tasks and the graded response to cognitive workload levels confirm that these methods capture meaningful neural activity patterns even under less-than-ideal conditions. This validates the utility of source localization in research settings where individual structural imaging is unavailable or impractical. While our approach does not provide absolute validation of source localization accuracy, which would require ground truth data such as simultaneous intracranial recordings, it demonstrates that established methods produce neurophysiologically plausible results that align with established knowledge about brain function during visual processing and cognitive tasks.

Scholars interested in estimating sources within the brain are provided with some recommendations. The first is to employ an average brain for the specific population of interest (children, adult females, and adult males) because of the different anatomies, such as skull thickness or brain size. The second recommendation is to spend enough time aligning the components of the forward model, such as the electrodes and the fiducials, to minimize the systematic bias of the inverse solution. The third recommendation is to design experiments robustly by acknowledging that EEG signals are inherently noisy, even if preprocessed and cleaned and therefore influence the source space estimation. Finally, researchers should be careful when interpreting results, given the many configurations of the inverse model's parameters, such as the inverse method, regularization settings, and conductivity values, which will lead to different results. There exists systematic bias within these methods and so it's better to analyze differences between source space activation rather than the overall source space. Eventually, because of the nature of the inverse problem, an inverse method only generates estimations. It does not determine the brain activation that led to a particular set of EEG recordings since infinitely many configurations can exist.

## 7 Conclusion

This study has demonstrated that effective EEG pre-processing and source localization can be achieved without subject-specific anatomical data through a fully automated pipeline. The successful validation across naturalistic viewing and controlled cognitive tasks suggests broad applicability across different experimental contexts. However, several important directions for future research emerge from this work.

While the current method performs well with adult participants, extending its application to pediatric populations will require incorporating age-appropriate head models and validation against developmental changes in brain structure and function. Furthermore, the observed relationship between regional size and activation differences indicates a need for more sophisticated spatial normalization approaches to account for anatomical variability across subjects and regions.

While individual anatomical variations can influence source localization accuracy, using standardized head models remains a viable approach for many research applications. Our validation across two independent datasets demonstrates consistent and interpretable activation patterns, suggesting that template-based source localization can effectively capture meaningful neural activity patterns. This finding aligns with previous research by Valdés-Hernández et al. (2009), who showed that template-based approaches could achieve localization accuracies comparable to individual MRI-based solutions in many cases. Similarly, Song et al. (2015) demonstrated that standardized head models can provide reliable source estimates when individual MRI data is unavailable. Our approach builds upon these established findings while addressing practical constraints often encountered in research settings, such as limited access to MRI facilities, cost considerations, and time constraints. The robust performance observed across our demographically diverse datasets supports the utility of this standardized approach, particularly in contexts where individual structural imaging is not feasible or practical.

It is important to note that the optimal choice of pre-processing methods remains an open question in the field. As highlighted in recent discussions (Delorme, 2023), automatic artifact removal and other pre-processing strategies continue to be debated. In our work, we aimed to maximize the signal-to-noise ratio, assuming that improved SNR would lead to better source localization accuracy; however, the precise impact of each pre-processing step on localization performance has not been fully elucidated and will require further investigation.

Additionally, the pipeline's parameters—including grid size, BEM resolution, and regularization—were selected based on preliminary testing and the existing literature. It is conceivable that alternative configurations might yield enhanced performance for specific tasks, and systematic exploration of these parameters represents an important direction for future work.

The open-source availability of the pipeline as a Python package facilitates reproducibility and invites further development by the research community. This accessibility, combined with the pipeline's automation, makes it a valuable tool for researchers seeking to incorporate EEG source localization into their analyses without requiring manual preprocessing or multimodal data. The primary contribution of this work is not a novel source localization method but rather an accessible Python implementation that incorporates modern head models and atlases with established methods, optimized for naturalistic EEG data without requiring subject-specific information. While software like LORETA-KEY, FieldTrip, Brainstorm, and EEGLAB provide similar functionality, this pipeline offers distinct advantages for researchers working in the Python ecosystem who require a streamlined approach for analyzing mid-length naturalistic EEG data.

In conclusion, this work represents a significant step toward making EEG source localization more accessible and practical for real-world applications while maintaining scientific rigor. The successful validation across different experimental paradigms suggests that the proposed approach could be valuable in research and clinical settings. Future work addressing pre-processing uncertainties, different head geometries, and optimizing parameter configurations will further enhance the robustness and applicability of the proposed method.

## Data Availability

The data that support the findings of this study are publicly available. Healthy Brain Network, which contains EEG signals, can be accessed at: https://data.healthybrainnetwork.org/main.php. ICBM 2009c Nonlinear Symmetric and the Cerebra Atlas are publicly accessible at: https://nist.mni.mcgill.ca/icbm-152-nonlinear-atlases-2009/.
