# Supplementary material for: Evaluation of EEG pre-processing and source localization in ecological research
Source: Front Neuroimaging. 2025 Mar 31;4:1479569. doi: 10.3389/fnimg.2025.1479569 (PMC11994696; doi:10.3389/fnimg.2025.1479569)
Supplement: Supplementary file 1 [file Data_Sheet_1.pdf]

Supplementary Material

1 PIPELINE DESIGN DETAILS

1.1 Automatic EEG pre-processing

A1 - Downsampling - The first preprocessing step involves downsampling the data. Downsampling involves removing data points to facilitate subsequent computations. This resampling might seem counter-intuitive since the data might be subsequently used for artefact removal via decomposition (example with ICA). However, as pointed out by Makoto, it is not the number of data points that dictates the quality of a decomposition but rather the total recording time. As a general rule of thumb, it is suggested to select a resampling rate that is double the highest frequency of interest, following the Nyquist theorem.

A2 - Bandpass filtering - After downsampling, a band pass filter removes unwanted frequencies unrelated to neural activity. EEG signals typically range from 0.1 to 100 Hz, with most clinically relevant activity occurring below 50 Hz (Teplan et al., 2002).

A3 - PREP strategy - After filtering, the standardized early-stage EEG processing pipeline (PREP) (Bigdely-Shamlo et al., 2015) is is adopted to automatically remove 'bad channels'. This automatically detects bad channels and their interpolation with good ones around them. Since the new interpolated channel is a linear combination of surrounding electrodes, it gets discarded in a later ICA whitening step. Additionally, the PREP pipeline includes a systematic approach for removing line noise, typically 50 or 60 Hz, and their harmonics. Finally, considering the interpolated channels, the data is re-referenced to the 'true' average reference, accounting for the 'bad channels.'

A4 - Automatic ICA component removals - The PREP pipeline is followed by an automatic ICA component removal step, eliminating unwanted signal components based on their statistical properties in the time and frequency space. This process is depicted in figure S1. This starts by executing Principal Component Analysis (PCA) on the multivariate signal space. The principal components that explain at least a significant amount of the variance are passed onto the ICA step as part of signal whitening. For example LeVan et al. (2006) set this value to 90%. This pre-whitening phase helps transform signals, coming from the previous phase (A3) into PCA components, which are reduced in number, according the threshold mentioned above, for removing their linear combinations. This is particularly important given the previous channel interpolation step made the signal rank deficient. Once the ICA components have been identified, unwanted components are removed automatically, based on Nolan et al. (2010) and Raufi and Longo (2022). Specifically, anomalous components are discarded based on spectral kurtosis, Hurst exponent, mean power density slope and median component slope. In detail, components with a z-score greater than ± 3 in at least one of these four dimensions are discarded. Signals in the scalp space's original time domain are reconstructed with the inverse ICA. Signals are then remixed without the excluded components, including those identified in the PCA step and not passed onto ICA. The reconstructed signals (figure **??** A4)) obtained with inverse ICA are the output of the pre-processing pipeline and the input of the source localization phase, as described in the next section. This output should have a greater signal-to-noise ratio, given that some artefacts should have been removed and the neural signal preserved. After pre-processing, a meaningful cleaner signal can be injected into the source localization methodology to obtain source estimates indicating the brain's spatial activation over time

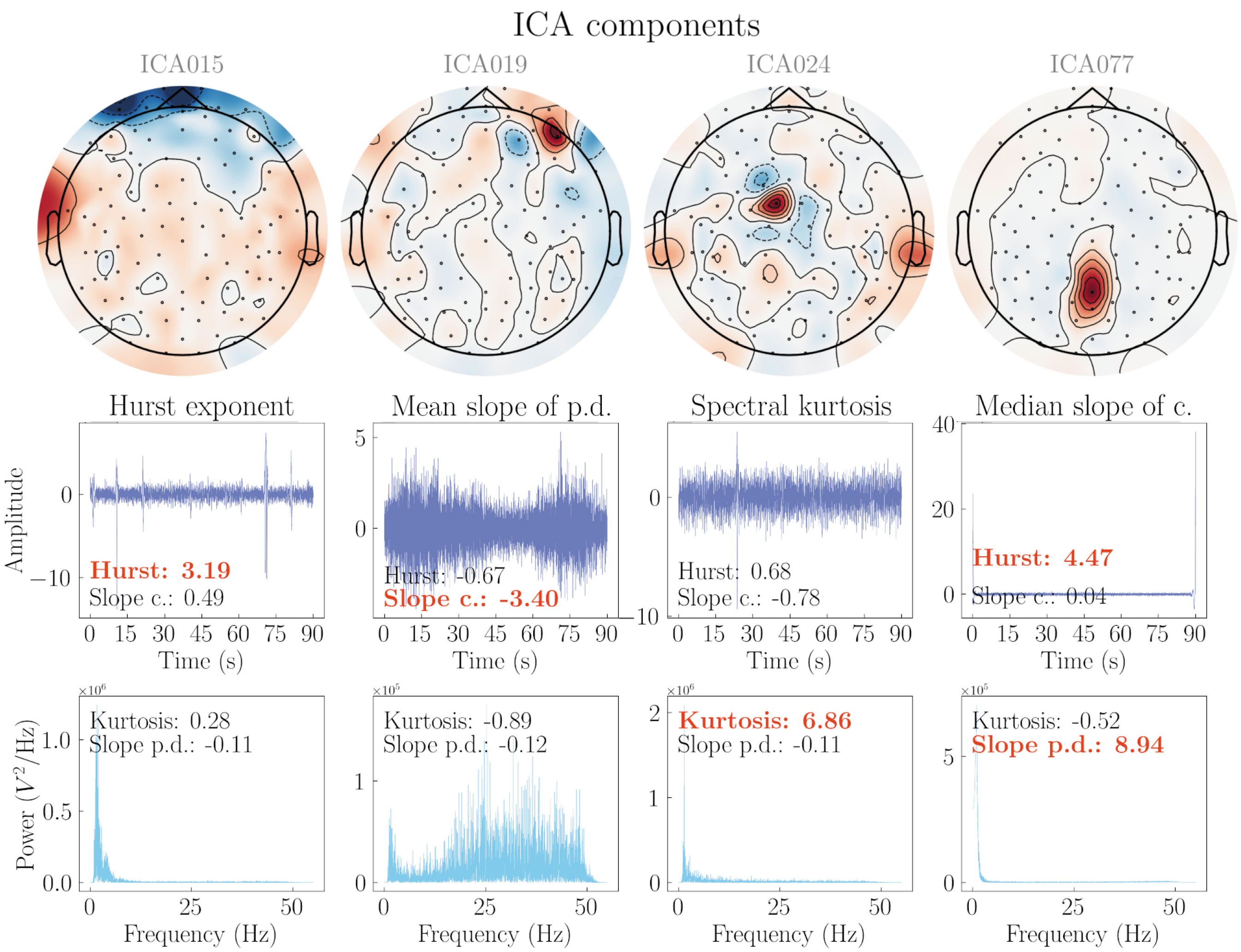

Figure S1. A4 - Automatic ICA artefact identification and correction pre-processing step for subject's NDARRA733VWX resting state data from the Healthy Brain Network dataset (same as figure **??**). The top row shows 4 topo-maps associated with the removed components. Each plot represents the overall mean activation for each particular component. The second row is the projection of the component plotted against time, and the bottom row is the power spectrum of the component. Each column represents components removed because of having z-scores $\pm$ 3 for their Hurst exponent, the median slope of the component, spectral kurtosis, and the mean slope of the power density, respectively.

1.2 Source localization

The forward model describes how electrical currents generated by neural sources propagate through the head to produce the potentials measured at the scalp. It can be represented mathematically as:

$$\mathbf{v} = \mathbf{L} \cdot \mathbf{j} + \mathbf{n} \tag{S1}$$

where $\mathbf{v}$ is the vector of measurements at the sensors, $\mathbf{L}$ is the lead field matrix, $\mathbf{j}$ is the vector representing the source distribution, and $\mathbf{n}$ represents measurement noise. The forward model consists of three main components:

- Boundary Element Model (BEM)
- Source Space

- Electrode Montage

The BEM represents the head as a set of nested surfaces with different electrical conductivities, typically including the brain, skull, and scalp. The BEM is crucial for accurately modeling the propagation of electrical fields through the head tissues. The brain-to-skull conductivity ratio (BSCR) is a critical parameter in the BEM, with reported values ranging from 8 to 80 (Akalin Acar and Makeig, 2013). The source space defines the possible locations of neural sources within the brain. It is typically represented as a set of dipoles with known positions and unknown orientations and magnitudes. The choice of source space affects the spatial resolution and computational complexity of the source localization problem. The electrode montage specifies the positions of the EEG electrodes on the scalp. In ideal scenarios, precise digitized electrode positions would be available for each recording. However, standardized electrode positions based on the International 10-20 system or its extensions are used in many practical applications.

The inverse problem in EEG source localization involves estimating the neural sources $\mathbf{j}$ given the measured scalp potentials $\mathbf{v}$ and the lead field matrix $\mathbf{L}$. The inverse problem is ill-posed, as infinitely many possible source configurations could produce the observed scalp potentials. Various inverse methods exist, each with its assumptions and constraints. The general form of the inverse solution can be expressed as:

$$\mathbf{j} = \mathbf{W} \cdot \mathbf{v} \tag{S2}$$

where $\mathbf{W}$ is the inverse operator. The challenge lies in determining an appropriate $\mathbf{W}$ that produces physiologically plausible and stable solutions. Several inverse methods have been developed to solve the EEG source localization problem.

1.2.1 B1 - Forward model

$B1_0$ **- Volume preparation**
Template selection
A standardized representation of brain anatomy is required to develop a subject-independent source localization methodology. For this purpose, the MNI-ICBM152-2009c Nonlinear Symmetric template (Fonov et al., 2009) is proposed as an average T1w MRI scan (figure S2 left). This template is derived from high-resolution MRI scans of many individuals and provides an average, normalized representation of brain anatomy. The MNI-ICBM152-2009c template combines high spatial resolution with a good signal-to-noise ratio, making it an ideal choice. It was generated through an iterative process where individual native MRIs were non-linearly fitted to the average template from the previous iteration, beginning with the MNI152 linear template (Fonov et al., 2011). This process ensures that the final template is not biased towards any single brain anatomy. In addition to the T1w MRI, the template provides complementary scans such as Gray matter, White matter, and Cerebrospinal fluid maps, which are valuable for accurate tissue segmentation and modelling. To enhance the anatomical precision of the source space, the CerebrA atlas (Manera et al., 2019) is integrated with the MNI-ICBM152-2009c template (figure S2 right). The CerebrA atlas is the human brain's high-resolution probabilistic anatomical atlas, specifically tailored to analyze functional and structural neuroimaging data. It provides detailed information about the brain's anatomical structures, including cortical and subcortical regions, white matter tracts, and cerebrovascular structures. The CerebrA atlas is aligned with the MNI-ICBM2009c coordinate system, which allows for easy overlay of the anatomical information onto the MNI-ICBM2009c standard space. This integration provides a more accurate and detailed parcellation of brain regions, which is crucial for precise source localization.

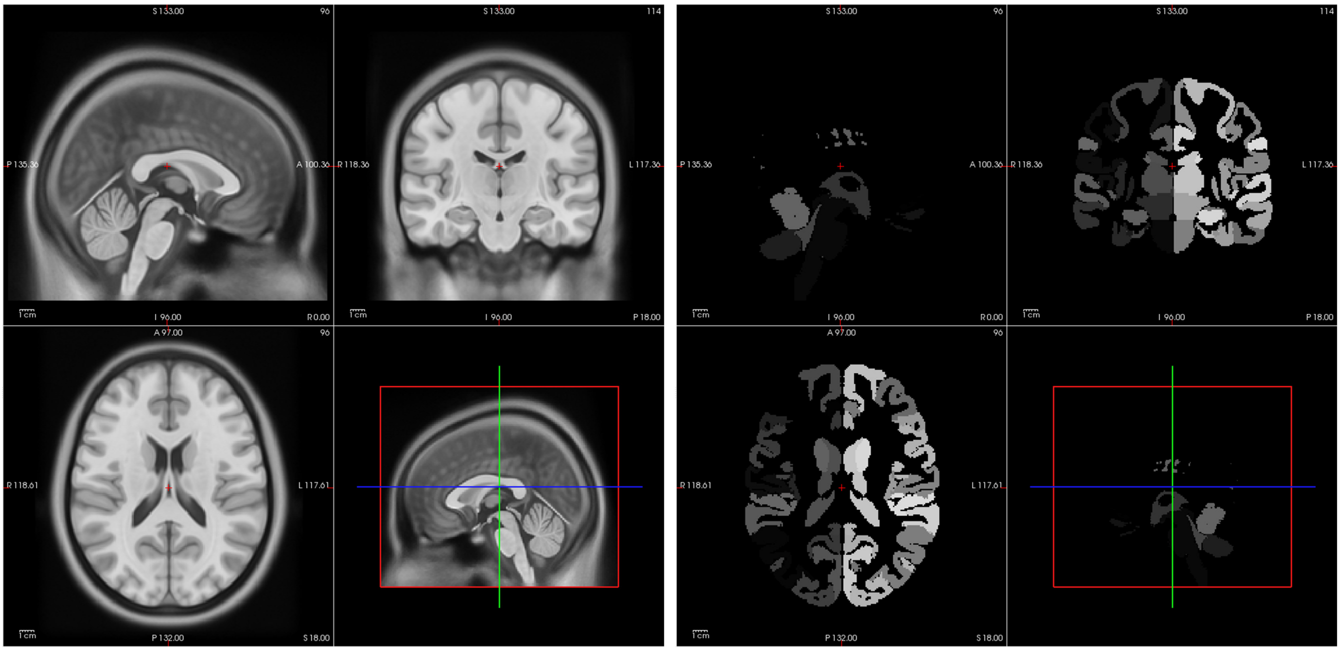

ICBM_2009c_Nonlinear_Symmetric/
mni_icbm152_nlin_sym_09c_nifti/
mni_icbm152_t1_tal_nlin_sym_09c.nii

CerebrA.nii

Figure S2. MNI-ICBM2009c Nonlinear Symmetric t1w MRI (left) and CerebrA atlas (right)

Cortical reconstruction

Cortical reconstruction allows for accurate modelling of the complex geometry of the brain's cortical surface. This process is essential for generating a realistic source space and for accurately modelling the propagation of electrical fields through the head tissues. FreeSurfer, a widely-used and well-validated software suite for cortical reconstruction and volumetric segmentation (Fischl, 2012) is adopted to perform this step. FreeSurfer employs automated algorithms to process the MRI data, including intensity normalization, Skull stripping, White matter segmentation, Surface generation and topology correction, and Pial surface generation. This process's output includes detailed surface meshes representing the white matter and pial surfaces and volumetric segmentations of various brain structures. These outputs are crucial for generating the source space and creating an accurate Boundary Element Model (BEM) for the forward solution. FreeSurfer's cortical reconstruction on the MNI-ICBM152-2009c template ensures the process is based on a standardized, high-quality representation of cortical anatomy. This approach generates a consistent source space across different subjects, which is essential for subject-independent source localization.

Coordinate frame alignment

One of the challenges in integrating the CerebrA atlas with the MNI-ICBM152-2009c template is that they use different coordinate systems and have different dimensions (figure S3). To address this, a coordinate frame alignment step is performed to ensure that the CerebrA atlas and the T1w MRI scan are in the same space. The T1w MRI scan is a $[256, 256, 256]$ voxel volume in the Left-Inferior-Anterior (LIA) coordinate frame. In contrast, the CerebrA volume is a $[193, 229, 193]$ voxel grid in the Right-Superior-Anterior (RAS) coordinate frame. Each volume has its affine matrix, which describes the transformation from voxel coordinates to world coordinates. FreeSurfer's $mri_vol2vol$ utility aligns these coordinate frames. This tool allows for resampling the CerebrA atlas into the same space as the T1w MRI. After this process, both volumes share the same affine matrix and coexist within a $[256, 256, 256]$ volume. This alignment step is

crucial for ensuring that the anatomical information from the CerebrA atlas accurately corresponds to the structural information in the T1w MRI. It allows for precise mapping of the detailed parcellation of brain regions from the CerebrA atlas onto the source space, enhancing the anatomical accuracy of the source localization methodology.

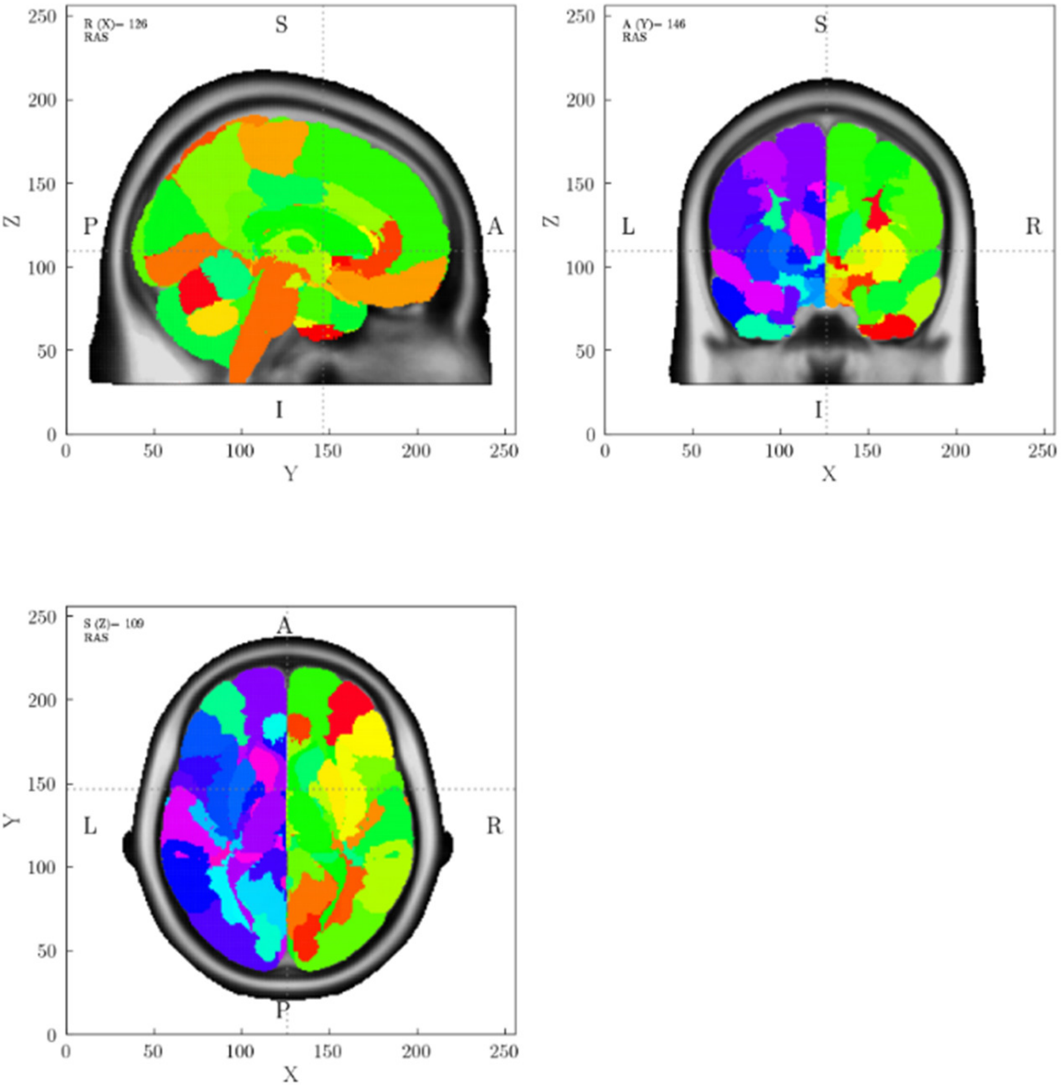

Figure S3. Coordinate frame alignment between the MNI-ICBM2009c Nonlinear Symmetric t1w MRI and the CerebrA The proposed pipeline incorporates an automatic EEG pre-processing strategy, implements source localization by employing a forward model based on the average ICBM T1-weighted MRI scans in conjunction with the CerebrA atlas and standard electrode positions, and applies inverse modeling through the eLORETA algorithm. A three-layer Boundary Element Method (BEM) model, derived from these average MRI scans, integrates standard electrode positions and a volumetric source space from the CerebrA atlas to construct this common forward model. No participant-specific data is considered. A shared forward model is derived from the average T1 MRI scans, the CerebrA atlas, and standard electrode positions.atlas volume. The shared affine is plotted as a dotted line. Right-Anterior-Superior (RAS) coordinate frame. $[256, 256, 256]$ volume.

Region metadata cleanup

After aligning the CerebrA atlas with the MNI-ICBM152-2009c template, the resulting volume is analyzed

to extract valuable metadata about the brain regions. This information is crucial for understanding the distribution of the source space and for interpreting the source localization results. The aligned CerebrA volume contains 1,169,569 voxels, representing 6.97% of the total $[256, 256, 256]$ grid. Of these, 850,933 voxels (5.08%) are categorized as cortical regions, and 318,636 voxels (1.89%) are categorized as non-cortical regions. The atlas comprises 102 unique regions, evenly split between the right and left hemispheres (51 regions each). 62 are labelled cortical, and 40 are labelled non-cortical. This detailed parcellation allows for a fine-grained analysis of brain activity. The regions vary significantly in size, which is essential to consider when interpreting source localization results. The smallest area is the basal forebrain, comprising only 0.04% of the total atlas volume (combining both hemispheres). In contrast, the Cerebellum Gray Matter is the most significant overall region, with a combined hemisphere density of 12.18%. The Superior Frontal region is the largest cortical area, with a density of 9.04% (see table S1).

$B1_1$ **- BEM layers**
The Boundary Element Model (BEM) is a crucial The proposed pipeline incorporates an automatic EEG pre-processing strategy, implements source localization by employing a forward model based on the average ICBM T1-weighted MRI scans in conjunction with the CerebrA atlas and standard electrode positions, and applies inverse modeling through the eLORETA algorithm. A three-layer Boundary Element Method (BEM) model, derived from these average MRI scans, integrates standard electrode positions and a volumetric source space from the CerebrA atlas to construct this common forward model. No participant-specific data is considered. A shared forward model is derived from the average T1 MRI scans, the CerebrA atlas, and standard electrode positions.component of the forward model, as it represents the different tissue layers of the head and their electrical properties. A 3-layer BEM model is adopted, which includes the brain, skull, and scalp. This model is generated using the watershed algorithm (Romero-Zaliz and Reinoso-Gordo, 2018) implemented in FreeSurfer. One of the challenges encountered in generating the BEM was ensuring that the inner skull surface was entirely contained within the outer surfaces (see supplementary material). To address this, manual editing using Blender software was performed to adjust the innermost layer where necessary. The conductivity values assigned to each layer of the BEM are critical for accurately modelling the propagation of electrical fields through the head.

It is worth noting that the literature does not universally agree on the choice of the brain-to-skull conductivity ratio (BSCR). Some studies have reported better accuracies with a smaller ratio (1:20) (Wang and Ren, 2013; Acar et al., 2016), while others have found improved performance with larger ratios. However, it is generally accepted that a high BSCR (1:80) tends to result in more outward-oriented source estimates (Akalin Acar and Makeig, 2013). Another important consideration is the resolution of the BEM surfaces. In this implementation, each of the three BEM surfaces contains 5,120 triangles. This level of detail provides a good balance between accuracy in representing the head geometry and computational efficiency. The choice of BEM resolution, like the source space density, represents a trade-off between model accuracy and computational complexity. A higher resolution BEM can more accurately represent the head geometry, potentially leading to more accurate forward solutions. However, it also increases the computational demands of the forward model calculation. The choice of 5,120 triangles per surface provides a good compromise between these factors, allowing for accurate modelling of the head tissues while maintaining reasonable computational requirements.

$B1_2$ **- Source space generation**
The source space is a critical component of the EEG source localization methodology, as it defines the possible locations of neural sources within the brain. This approach generates the source space using the cortical regions defined in the CerebrA atlas. This decision is based on several important considerations.

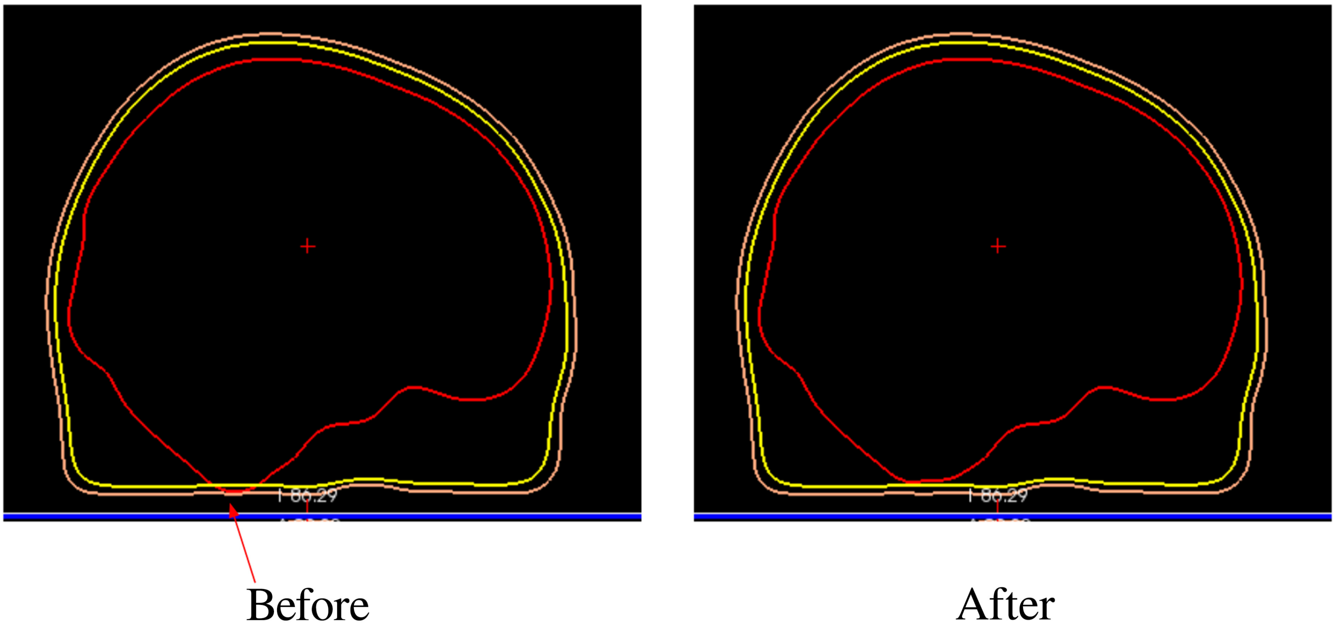

Figure S4. BEM manual editing by squeezing the inner layer so that it is contained within the outer layers.

EEG is primarily sensitive to the electrical activity generated by neurons in the cortex due to its proximity to the scalp (Courellis et al., 2017). Focusing on cortical regions allows for a more manageable and accurate model (Fanciullacci et al., 2021). The cortex's layered structure is conducive to generating the synchronized neural activity detected by EEG. The source space generation process involves downsampling the cortical regions of the CerebrA atlas. An equally spaced grid is created, where the spacing between grid points is controlled by a parameter g_s (grid size). The choice of g_s represents a trade-off between spatial resolution and computational complexity (figure S5). A smaller g_s value results in a denser source space with more potential source locations, potentially improving spatial resolution but increasing computational demands. A larger g_s value produces a sparser source space, reducing computational complexity at the cost of lower spatial resolution.

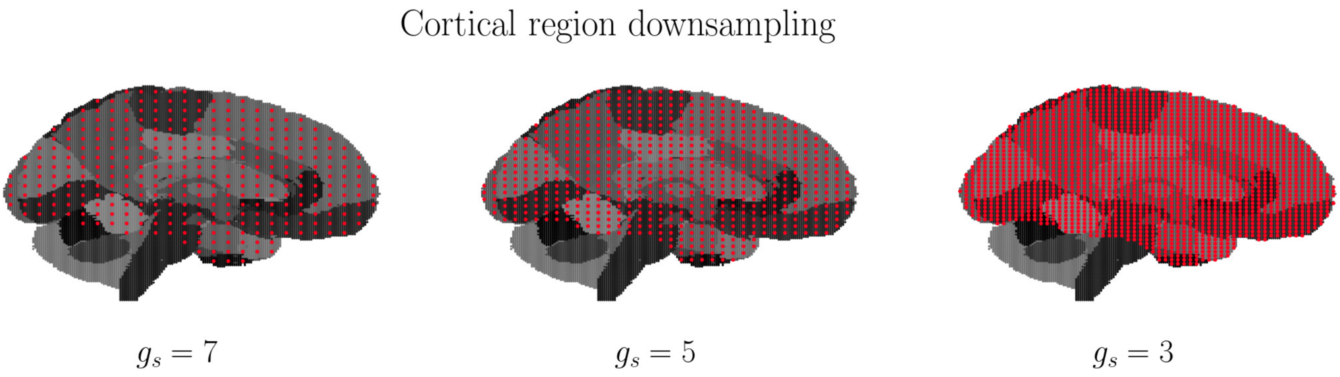

Figure S5. Source space generation from CerebrA cortical regions. Setting a different g_s value results in a different source space density. The amount of sources for g_s = 7,5 and 3 are 2,541, 6,580, and 31,553, respectively.

Given three different g_s values, a varying number of sources can be generated:

- $g_s = 7$ results in 2,541 sources
- $g_s = 5$ results in 6,580 sources
- $g_s = 3$ results in 31,553 sources

It's important to note that while a denser source space can theoretically provide better spatial resolution, it also increases the inverse problem's ill-posedness. This is because the number of potential sources greatly exceeds the number of EEG sensors, making the problem more underdetermined. The choice of source space density aims to strike a balance between these competing factors. The resulting source space provides a detailed representation of potential neural sources across the cortex, with the density of sources varying according to the size and shape of different cortical regions as defined in the CerebrA atlas. This anatomically informed approach to source space generation enhances the physiological plausibility of the source localization results.

$B1_3$ **- Electrode montage**

The final component of the forward model is the electrode montage, which specifies the positions of the EEG electrodes on the scalp (see supplementary figures). In an ideal scenario, precise, digitized electrode positions would be available for each recording. However, such detailed information may not be available in many real-world applications, particularly those involving portable EEG systems. To address this, the proposed pipeline uses standardized electrode positions based on widely used EEG montages, such as the International 10-20 system or its extensions. This approach makes the proposed pipeline flexible and applicable to a wide range of EEG recording setups. A parameter h_s (head size) is introduced that allows for some adjustment of the electrode positions to account for variations in head size. This parameter determines the scaling of the distances between electrodes. A critical step in incorporating the electrode montage into the forward model is aligning it with the BEM surfaces. This process involves two main components:

- **Fiducials:** These reference points align the electrode positions with the head model. Typically, the nasion and left and right preauricular points are used as fiducials.
- **Head-MRI transformation:** This is a transformation matrix that maps between the coordinate system of the electrode positions and that of the MRI/BEM (See figure S6).

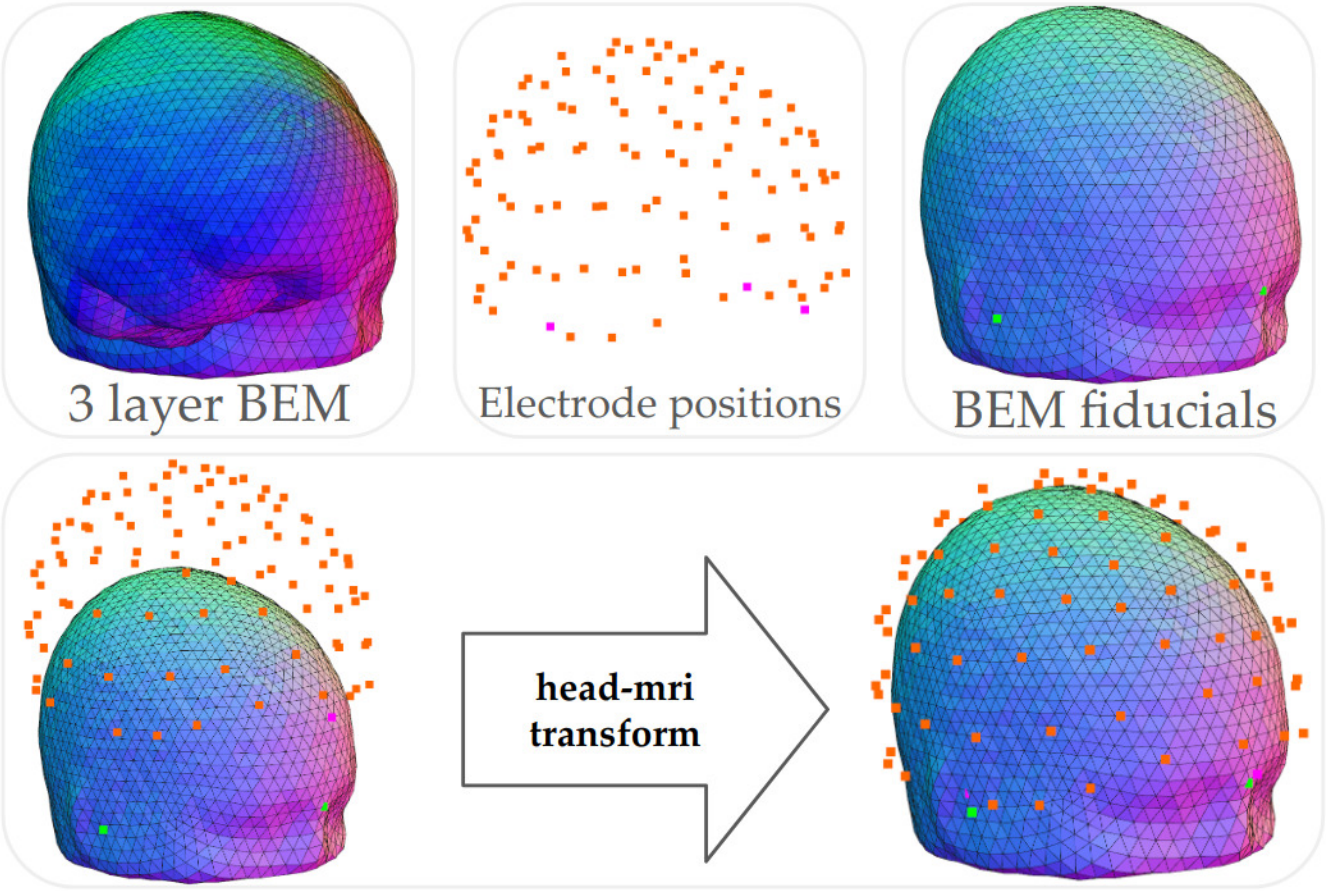

Figure S6. BEM fiducials and head-mri transformation by manually generated adjusting for best fit.

MNE-Python's coregistration tool [1] manually determines the fiducials and head-MRI transformation, adjusting for the best fit between the electrode positions and the BEM surfaces. This step ensures that the electrode positions in the forward model accurately correspond to their intended locations on the scalp surface of the head model. The pipeline maintains flexibility while allowing accurate forward modelling by using standardized electrode montages and providing a mechanism for adjusting to different head sizes. This approach is particularly suitable for applications in naturalistic settings where precise electrode position information may not be available for each recording.

$B1_4$ **- Merging components**
Eventually, with all the components in place - the BEM, source space, and electrode montage - the forward model can be constructed. Such a model, represented by the lead field matrix $\mathbf{L}$, describes how unit dipole sources at each location in the source space would project to the scalp electrodes.

1.3 Summary of Pipeline parameters

The proposed pipeline, in synthesis, involves setting key parameters that can be adjusted based on the specific requirements of a study or the characteristics of the EEG data being analyzed:

- $B1_1$) BEM conductivity ratios (b_c): inner skull, outer skull, and outer skin for determining the damping of the currents; and BEM resolution (b_r): number of triangles in the BEM layer;

[1] https://mne.tools/stable/index.html

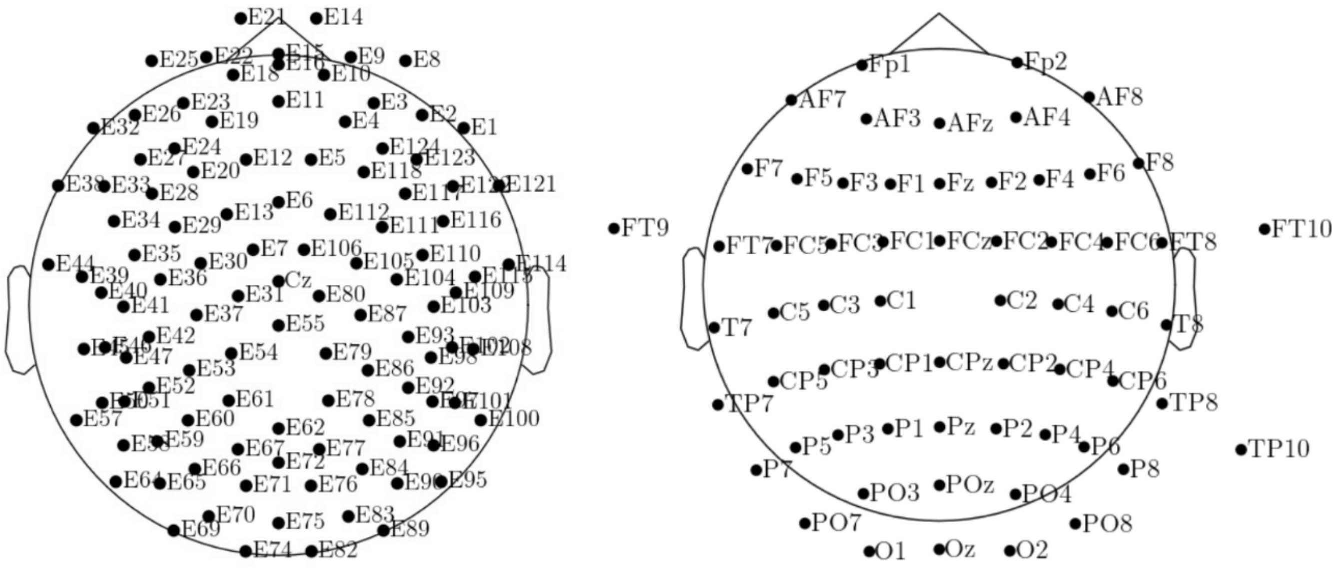

Figure S7. Left: HBN montage, GSN-HydroCel montage downsamples to 109 electrodes by removing chin and neck electrodes. Right: COGBCI montage, 62 electrodes 10/20 system

- $B1_2$) Source space grid size (g_s): the number of points (density) of the source space;
- $B1_3$) Electrode positions (e_p): produced by considering head size (h_s) and electrode standard montage (e_m).
- $B2$) Inverse method (i_m): heuristic for determining source space activation, given electrode-level recordings;

These parameters provide flexibility in the pipeline, allowing it to be adapted to different experimental setups and research questions. However, it is important to note that the optimal values for these parameters may vary depending on the specific application and the characteristics of the EEG data being analyzed. Further testing and validation across various datasets and experimental conditions would be valuable for refining these parameter choices and understanding their impact on source localization accuracy. The pipeline's flexibility, particularly regarding adjustable parameters like source space density and electrode montage, allows it to adapt to different experimental requirements and EEG recording setups. This flexibility is crucial for ecological applications, where the specific conditions of EEG recording may vary. While the pipeline shows promise for subject-independent EEG source localization, it is important to acknowledge its limitations. Using a standardized head model cannot fully account for individual anatomical differences, which may affect the accuracy of source localization in some cases. However, it represents a good compromise if a generally applicable source localisation pipeline across people has to be implemented. Additionally, the pipeline's performance may vary depending on the quality of the EEG data and the specific cognitive processes being studied.

1.4 Pre-processing and source estimation parameters

Pre-processing strategy parameters -

- $A1$) Downsampling EEG data: 125Hz
- $A2$) Bandpass filtering: 1-50Hz

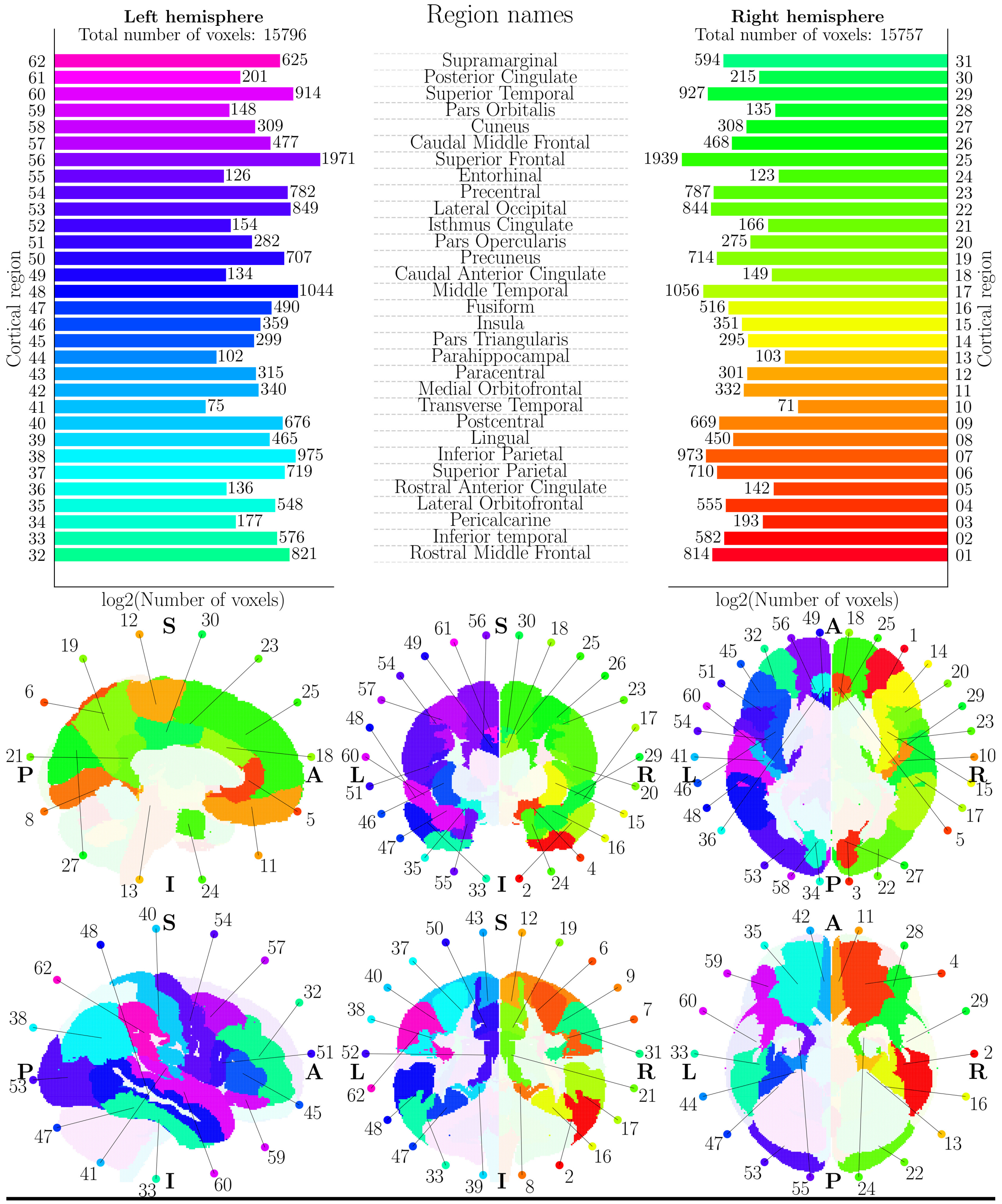

Figure S8. (TOP) Detailed bar chart representing the source space density, enumerating the number of voxels within the 62 cortical regions for the right and left hemispheres. Regions are colour-coded for easy identification, and the chart lists each region along with its corresponding cortical ID. The bar chart reveals the voxel distribution, offering insights into each cortical area's relative sizes and densities. (BOTTOM) Cortical regions are spatially represented in brain maps, showcasing their precise anatomical position within the cerebral cortex.

- $A3$) PREP pipeline: RANSAC enabled. 60Hz line noise removal. 8 iterations max. Not channel-wise. Not max chunk size.
- $A4$) Automatic ICA artifact correction: PCA variance threshold $>= 90\%$

Source localization parameters -

- $B1_1$) BEM conductivity ratios (b_c): inner skull=0.33S/m, outer skull=0.0042S/m, and outer skin=0.33S/m as suggested in with 1:80 conductivity ratio; BEM resolution (b_r): 5120 triangles per surface as suggested
- $B1_2$) Source space grid size (g_s): 3 (resulting in 31,553 sources);
- $B1_3$) Electrode positions: Head size (h_s): 0.1027, Electrode montage (e_m): GSN-HydroCel-129
- $B2$) Inverse method (i_m) eLORETA (with $\lambda^2 = \frac{1.0}{\text{SNR}^2}$);

An additional operation was the update of the original GSN-HydroCel-129 montage by removing unnecessary chin and neck electrodes resulting in 109 channels. Such resulting montage is manually lined up to the head model by generating BEM fiducials and the head-mri transformation (as described in section 1.2.1 using the MNE-co-registration tool), generating an inverse model. Such model is then used for all the subjects to estimate source activations.

2 RESULTS

2.1 Pre-processing effect on SNR and PSNR

For pre-processing evaluation, the signal-to-noise ratio (SNR) and the Peak signal-to-noise ratio (PSNR) are adopted; both metrics measure a signal's quality but focus on different aspects of the signal quality. SNR measures the ratio of a signal's power (meaningful information) to the power of background noise. In contrast, PSNR compares a signal's maximum possible power to the power of corrupting noise that affects its fidelity. SNR is a fundamental measure that quantifies the desired signal level relative to the background noise. A higher SNR indicates a clearer, more discernible signal, enhancing confidence in the detected neural activity. SNR is usually expressed in decibels (dB). SNR can be defined as the inverse of the coefficient of variation, i.e., the ratio of mean to standard deviation. Given an EEG signal with multiple channels, the signal-to-noise ratio (SNR) is computed using equation 2.1.

$$\text{SNR} = 10 \cdot \log_{10}\left(\frac{\overline{P}}{\sigma^2}\right) \tag{S3}$$

Similarly, PSNR is computed as:

$$\text{PSNR} = 10 \cdot \log_{10}\left(\frac{P_{\text{max}}}{\sigma^2}\right) \tag{S4}$$

where P_{max} is the maximum power of the signal and σ^2 is the variance of the signal power. Signal quality at every step of the pre-processing strategy can be measured by employing the signal-to-noise ratio (SNR) and peak signal-to-noise ratio (PSNR). A dependent parametric T-test can be used to measure the significance of the increase in quality of the signal in each pre-processing phase. Cohen's D can be used to measure the effect size of such increase.

Results demonstrate that the proposed automatic pre-processing pipeline significantly increases the signal's signal-to-noise ratio (SNR) and Peak Signal-to-noise ratio (PSNR) at each step. (Figure S9) The original raw data had an SNR of 55.49 ± 80.51, which increased to 80.51 ± 11.10 after applying a band-pass filter to the signal. The application of the PREP pipeline further improved such SNR to 82.52 ± 9.09. After artefact removal based on ICA, the SNR was 82.58 ± 10.45. PSNR values at each pre-processing step were as follows: 1) 78.19 ± 12.81, 2) 114.04 ± 7.35, 3) 115.52 ± 5.61 and 4) 114.52 ± 6.16. A dependent t-test was conducted at each preprocessing stage alongside the computation of Cohen's d. The findings indicate that applying a bandpass filter significantly enhanced the SNR and PSNR, as evidenced by a large effect size (SNR: $p < 0.001$, t = 18.0, d = 1.85; PSNR: $p < 0.001$, t = 31.17, d = 3.42). The PREP step was associated with a minor increase in SNR (p = 0.001, t = 3.35, d = 0.19) and PSNR ($p < 0.001$, t = 3.45, d = 0.22), indicating a small effect size. The last comparison revealed no significant change in SNR (p = 0.93) following the final Independent Component Analysis (ICA) artefact removal process, although an increment in signal quality was observed. For example, through visual inspection of the ICA components depicted in figure S1, it is possible to observe that some artefactual components were removed in phase A4 of the pre-processing strategy. However, there was a nominal decrease in PSNR ($p < 0.005$, t = 2.8, d = 0.16).

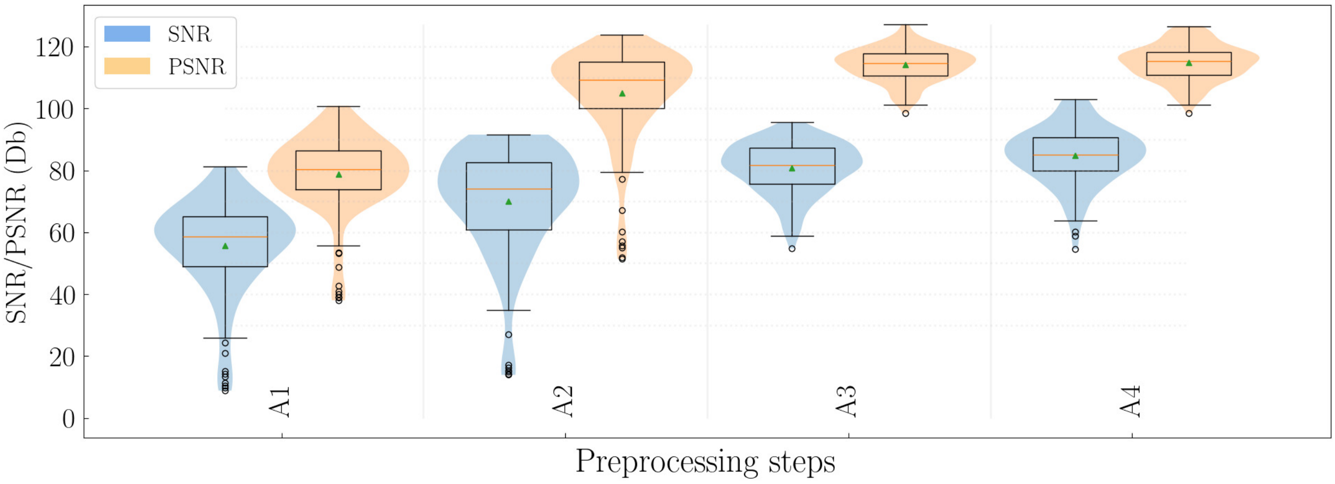

Figure S9. Signal-to-noise ratio and peak signal-to-noise ratio distributions for samples at each pre-processing step. 140 samples were analyzed in total: 35 EEG resting state samples and 105 video samples. SNR and PSNR significantly increased at every step. A1:Downsampling, A2: Bandpass filtering, A3: PREP pipeline, A4:Automatic ICA artefact correction)

Results demonstrate that the proposed automatic pre-processing pipeline also significantly increases the signal's signal-to-noise ratio (SNR) and Peak Signal-to-noise ratio (PSNR) at each step for the COG-BCI dataset. (Figure S10)

The original raw data had an SNR of 72.64 ± 6.77, which increased to 84.23 ± 5.59 after applying a band-pass filter to the signal. The application of the artifact removal based on ICA further improved the SNR to 89.62 ± 7.59.

PSNR values at each pre-processing step were as follows: 1) 89.09 ± 6.2, 2) 102.61 ± 5.26 and 3) 115.7 ± 4.91.

A dependent t-test was conducted at each pre-processing stage alongside the computation of Cohen's d. The findings indicate that applying a band-pass filter significantly enhanced the SNR and PSNR, as

evidenced by a large effect size (SNR: $p < 0.001$, t = 44.79, d = 1.91; PSNR: $p < 0.001$, t = 48.84, d = 2.34).

The last comparison revealed a significant increase in SNR (p ¡ 0.001, t = 24.46, d = 0.81) and PSNR ($p < 0.001$, t = 58.77, d = 2.56) following the final Independent Component Analysis (ICA) artifact removal process.

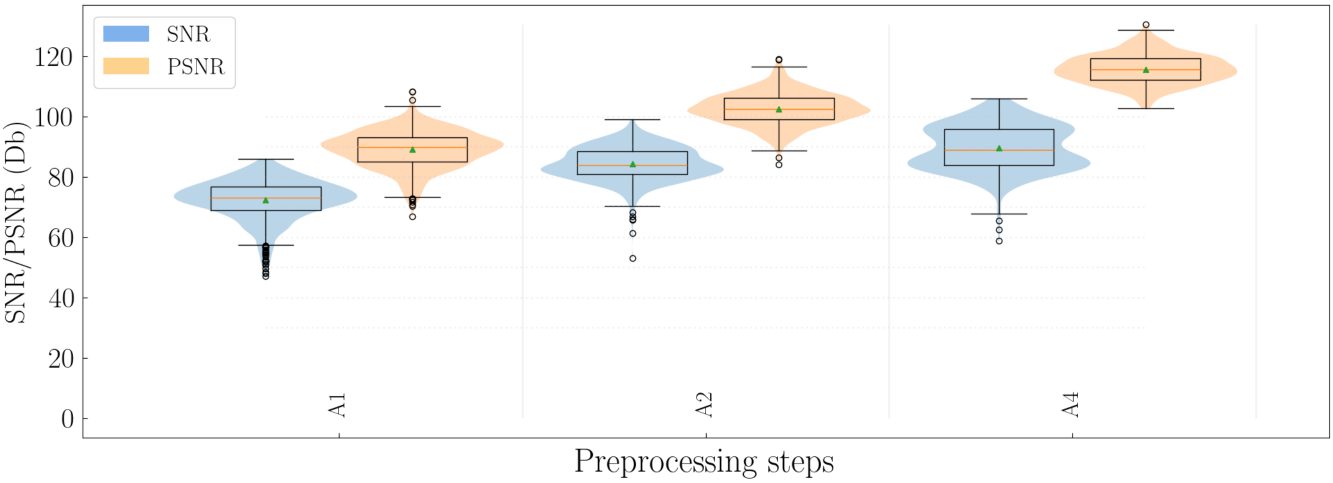

Figure S10. Signal-to-noise ratio and peak signal-to-noise ratio distributions for samples at each preprocessing step (COG-BCI dataset). 609 samples were analyzed in total: 3 sessions, 29 subjects, seven tasks each. SNR and PSNR significantly increased at every step. A1:Downsampling, A2: Bandpass filtering, A4:Automatic ICA artefact correction)

2.2 HBN

3 SUPPLEMENTARY DATA

Electrode names (GSN-HydroCel downsample to 109 electrodes. Remove chin and neck electrodes). [E1, E2, E3, E4, E5, E6, E7, E8, E9, E10, E11, E12, E13, E14, E15, E16, E18, E19, E20, E21, E22, E23, E24, E25, E26, E27, E28, E29, E30, E31, E32, E33, E34, E35, E36, E37, E38, E39, E40, E41, E42, E44, E45, E46, E47, E50, E51, E52, E53, E54, E55, E57, E58, E59, E60, E61, E62, E64, E65, E66, E67, E69, E70, E71, E72, E74, E75, E76, E77, E78, E79, E80, E82, E83, E84, E85, E86, E87, E89, E90, E91, E92, E93, E95, E96, E97, E98, E100, E101, E102, E103, E104, E105, E106, E108, E109, E110, E111, E112, E114, E115, E116, E117, E118, E121, E122, E123, E124, Cz]

Electrode names (10/20 to 62 electrodes.). [Fp1, Fz, F3, F7, FT9, FC5, FC1, C3, T7, CP5, CP1, Pz, P3, P7, O1, Oz, O2, P4, P8, TP10, CP2, CP6, T8, C4, FC2, FC6, FT10, F8, F4, Fp2, AF7, AF3, AFz, F1, F5, FT7, FC3, C1, C5, TP7, CP3, P1, P5, PO7, PO3, POz, PO4, PO8, P6, P2, CPz, CP4, TP8, C6, C2, FC4, FT8, F6, AF8, AF4, F2, FCz]

4 SUPPLEMENTARY TABLES AND FIGURES

REFERENCES

[Dataset] Makoto. Makoto's preprocessing pipeline. `https://sccn.ucsd.edu/wiki/Makoto's_preprocessing_pipeline` (????). [Online; accessed 14-February-2024].

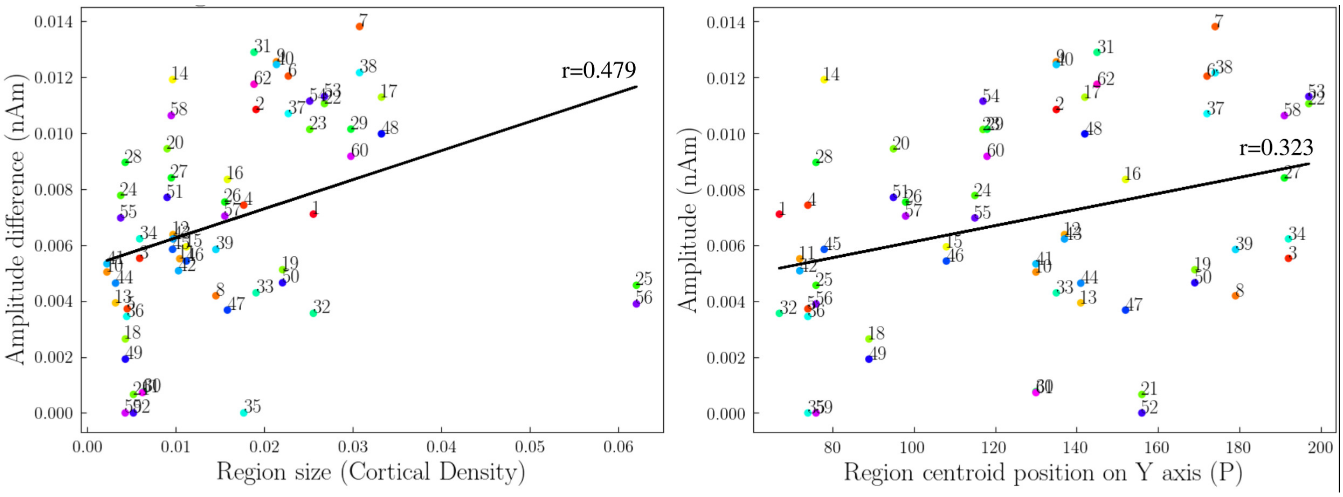

Figure S11. Left: Correlation ($r_s = 0.479, p < 0.001$) of the amplitude difference against cortical density, which is the % of the overall cortex's volume a particular region occupies. Right: Correlation ($r_s = 0.323, p = 0.010$) of the amplitude against region position on the Anterior-Posterior (Y) axis. Each dot is a value from a Cortical region ID (from 1 to 62). The regions with higher Y values are closer to the boundary of the voxel grid (with a max value of 256) at the back of the head (posterior). Contrarily, those regions with a centroid closer to 0 (opposite boundary) are closer to the front of the head (anterior).

Teplan M, et al. Fundamentals of eeg measurement. *Measurement science review* **2** (2002) 1–11.

Bigdely-Shamlo N, Mullen T, Kothe C, Su KM, Robbins KA. The prep pipeline: standardized preprocessing for large-scale eeg analysis. *Frontiers in neuroinformatics* **9** (2015) 16.

LeVan P, Urrestarazu E, Gotman J. A system for automatic artifact removal in ictal scalp eeg based on independent component analysis and bayesian classification. *Clinical neurophysiology* **117** (2006) 912–927.

Nolan H, Whelan R, Reilly RB. Faster: fully automated statistical thresholding for eeg artifact rejection. *Journal of neuroscience methods* **192** (2010) 152–162.

Raufi B, Longo L. An evaluation of the eeg alpha-to-theta and theta-to-alpha band ratios as indexes of mental workload. *Frontiers in Neuroinformatics* **16** (2022) 44.

Akalin Acar Z, Makeig S. Effects of forward model errors on eeg source localization. *Brain topography* **26** (2013) 378–396.

Fonov VS, Evans AC, McKinstry RC, Almli CR, Collins D. Unbiased nonlinear average age-appropriate brain templates from birth to adulthood. *NeuroImage* **47** (2009) S102.

Fonov V, Evans AC, Botteron K, Almli CR, McKinstry RC, Collins DL, et al. Unbiased average age-appropriate atlases for pediatric studies. *Neuroimage* **54** (2011) 313–327.

Manera AL, Dadar M, Fonov V, Collins DL. Cerebra: Accurate registration and manual label correction of mindboggle-101 atlas for mni-icbm152 template. *BioRxiv* (2019) 2019–12.

Fischl B. Freesurfer. *Neuroimage* **62** (2012) 774–781.

Romero-Zaliz R, Reinoso-Gordo J. An updated review on watershed algorithms. *Soft computing for sustainability science* (2018) 235–258.

Wang G, Ren D. Effect of brain-to-skull conductivity ratio on eeg source localization accuracy. *BioMed research international* **2013** (2013).

Acar ZA, Acar CE, Makeig S. Simultaneous head tissue conductivity and eeg source location estimation. *NeuroImage* **124** (2016) 168–180.

	Left hemisphere					Right hemisphere				
	CerebrA ID	Mindboggle ID	Name	Color	Density (%)	CerebrA ID	Mindboggle ID	Name	Color	Density (%)
Cortical	52	1027	Rostral Middle Frontal		1.86	1	2027	Rostral Middle Frontal		1.86
	54	1009	Inferior temporal		1.39	3	2009	Inferior temporal		1.39
	57	1021	Pericalcarine		0.43	6	2021	Pericalcarine		0.43
	58	1012	Lateral Orbitofrontal		1.29	7	2012	Lateral Orbitofrontal		1.29
	59	1026	Rostral Anterior Cingulate		0.32	8	2026	Rostral Anterior Cingulate		0.33
	60	1029	Superior Parietal		1.65	9	2029	Superior Parietal		1.65
	61	1008	Inferior Parietal		2.24	10	2008	Inferior Parietal		2.24
	63	1013	Lingual		1.06	12	2013	Lingual		1.06
	64	1022	Postcentral		1.56	13	2022	Postcentral		1.56
	65	1034	Transverse Temporal		0.16	14	2034	Transverse Temporal		0.16
	66	1014	Medial Orbitofrontal		0.75	15	2014	Medial Orbitofrontal		0.76
	67	1017	Paracentral		0.70	16	2017	Paracentral		0.70
	69	1016	Parahippocampal		0.23	18	2016	Parahippocampal		0.23
	73	1020	Pars Triangularis		0.70	22	2020	Pars Triangularis		0.70
	74	1035	Insula		0.81	23	2035	Insula		0.81
	75	1007	Fusiform		1.15	24	2007	Fusiform		1.15
	79	1015	Middle Temporal		2.42	28	2015	Middle Temporal		2.42
	81	1002	Caudal Anterior Cingulate		0.31	30	2002	Caudal Anterior Cingulate		0.31
	82	1025	Precuneus		1.60	31	2025	Precuneus		1.61
	83	1018	Pars Opercularis		0.66	32	2018	Pars Opercularis		0.66
	84	1010	Isthmus Cingulate		0.38	33	2010	Isthmus Cingulate		0.38
	85	1011	Lateral Occipital		1.95	34	2011	Lateral Occipital		1.95
	86	1024	Precentral		1.83	35	2024	Precentral		1.83
	87	1006	Entorhinal		0.27	36	2006	Entorhinal		0.27
	89	1028	Superior Frontal		4.52	38	2028	Superior Frontal		4.52
	93	1003	Caudal Middle Frontal		1.13	42	2003	Caudal Middle Frontal		1.13
	94	1005	Cuneus		0.69	43	2005	Cuneus		0.69
	95	1019	Pars Orbitalis		0.31	44	2019	Pars Orbitalis		0.31
	96	1030	Superior Temporal		2.17	45	2030	Superior Temporal		2.17
	98	1023	Posterior Cingulate		0.45	47	2023	Posterior Cingulate		0.46
	102	1031	Supramarginal		1.37	51	2031	Supramarginal		1.37
Non-cortical	53	631	Vermal lobules VI-VII		0.16	2	631	Vermal lobules VI-VII		0.20
	55	58	Accumbens Area		0.05	4	58	Accumbens Area		0.05
	56	44	Inferior Lateral Ventricle		0.09	5	44	Inferior Lateral Ventricle		0.09
	62	16	Brainstem		1.33	11	16	Brainstem		1.45
	68	85	Optic Chiasm		0.07	17	85	Optic Chiasm		0.07
	70	54	Amygdala		0.14	19	54	Amygdala		0.14
	71	632	Vermal lobules VIII-X		0.18	20	632	Vermal lobules VIII-X		0.22
	72	51	Putamen		0.57	21	51	Putamen		0.57
	76	92	Basal Forebrain		0.02	25	92	Basal Forebrain		0.02
	77	60	Ventral Diencephalon		0.52	26	60	Ventral Diencephalon		0.52
	78	52	Pallidum		0.15	27	52	Pallidum		0.15
	80	14	Third Ventricle		0.05	29	14	Third Ventricle		0.09
	88	15	Fourth Ventricle		0.05	37	15	Fourth Ventricle		0.07
	90	46	Cerebellum White Matter		1.11	39	46	Cerebellum White Matter		1.11
	91	49	Thalamus		0.86	40	49	Thalamus		0.86
	92	43	Lateral Ventricle		0.82	41	43	Lateral Ventricle		0.82
	97	45	Cerebellum Gray Matter		6.09	46	45	Cerebellum Gray Matter		6.09
	99	53	Hippocampus		0.40	48	53	Hippocampus		0.40
	100	50	Caudate		0.48	49	50	Caudate		0.48
	101	630	Vermal lobules I-V		0.34	50	630	Vermal lobules I-V		0.38

Table S1. Cerebra Atlas regions, with a unique identifier, mindboogle identifier, name, and density in percentage, compared to the whole brain volume.

Courellis H, Mullen T, Poizner H, Cauwenberghs G, Iversen JR. Eeg-based quantification of cortical current density and dynamic causal connectivity generalized across subjects performing bci-monitored cognitive tasks. *Frontiers in neuroscience* **11** (2017) 253493.

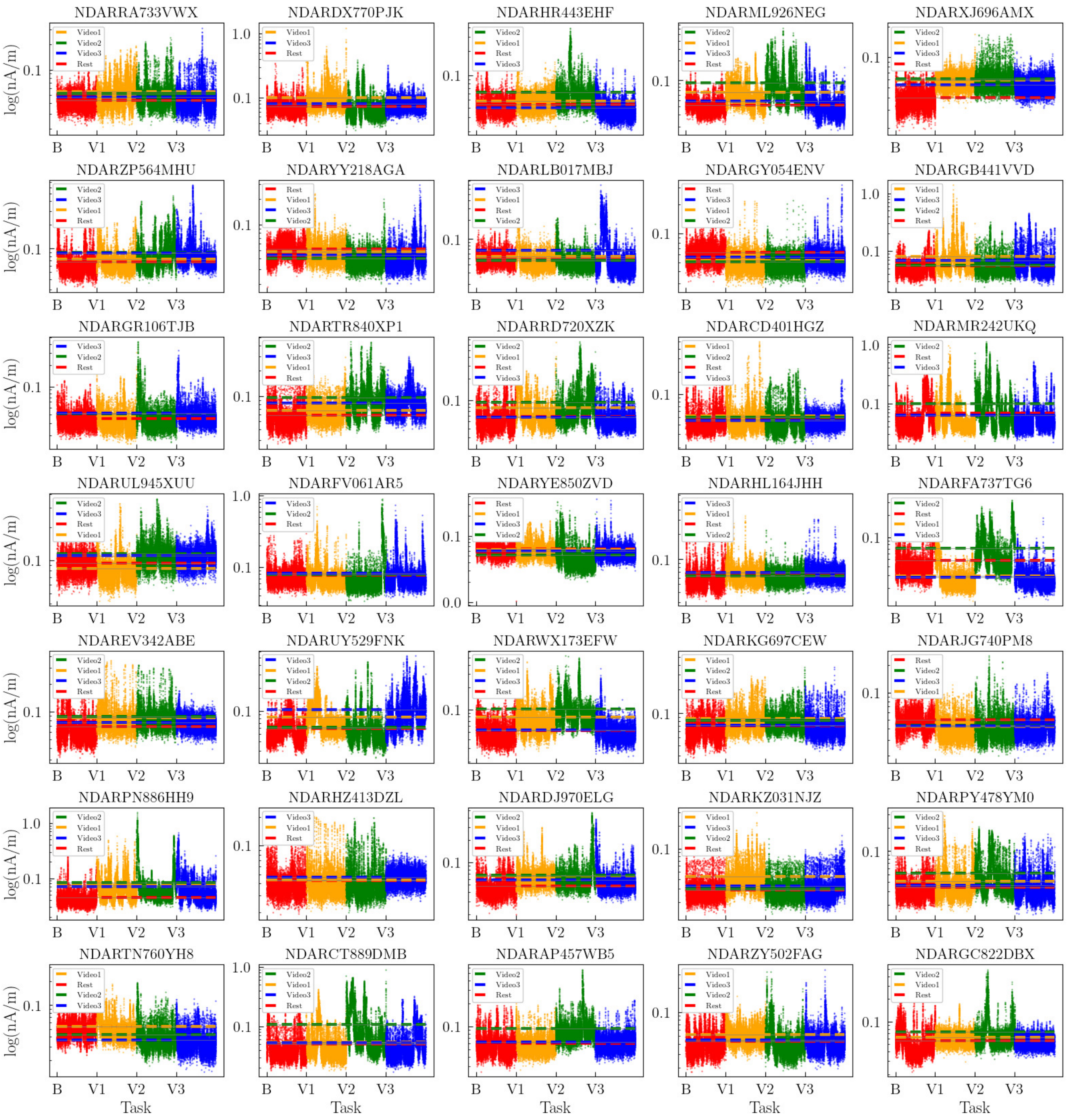

Figure S12. Within-subject findings with the mean source space activation for different conditions. Each plot represents a subject, and inside each plot, there are four colored blocks containing registered amplitudes in the source space activation matrix for each condition (B: Baseline(Rest), V1: Video 1, V2: Video 2, V3: Video 3). The legend is ordered so that the condition with the highest mean of its amplitudes is on top. If a video is not included in the legend, then the difference between the videos is not significant.

Fanciullacci C, Panarese A, Spina V, Lassi M, Mazzoni A, Artoni F, et al. Connectivity measures differentiate cortical and subcortical sub-acute ischemic stroke patients. *Frontiers in Human Neuroscience* **15** (2021) 669915.

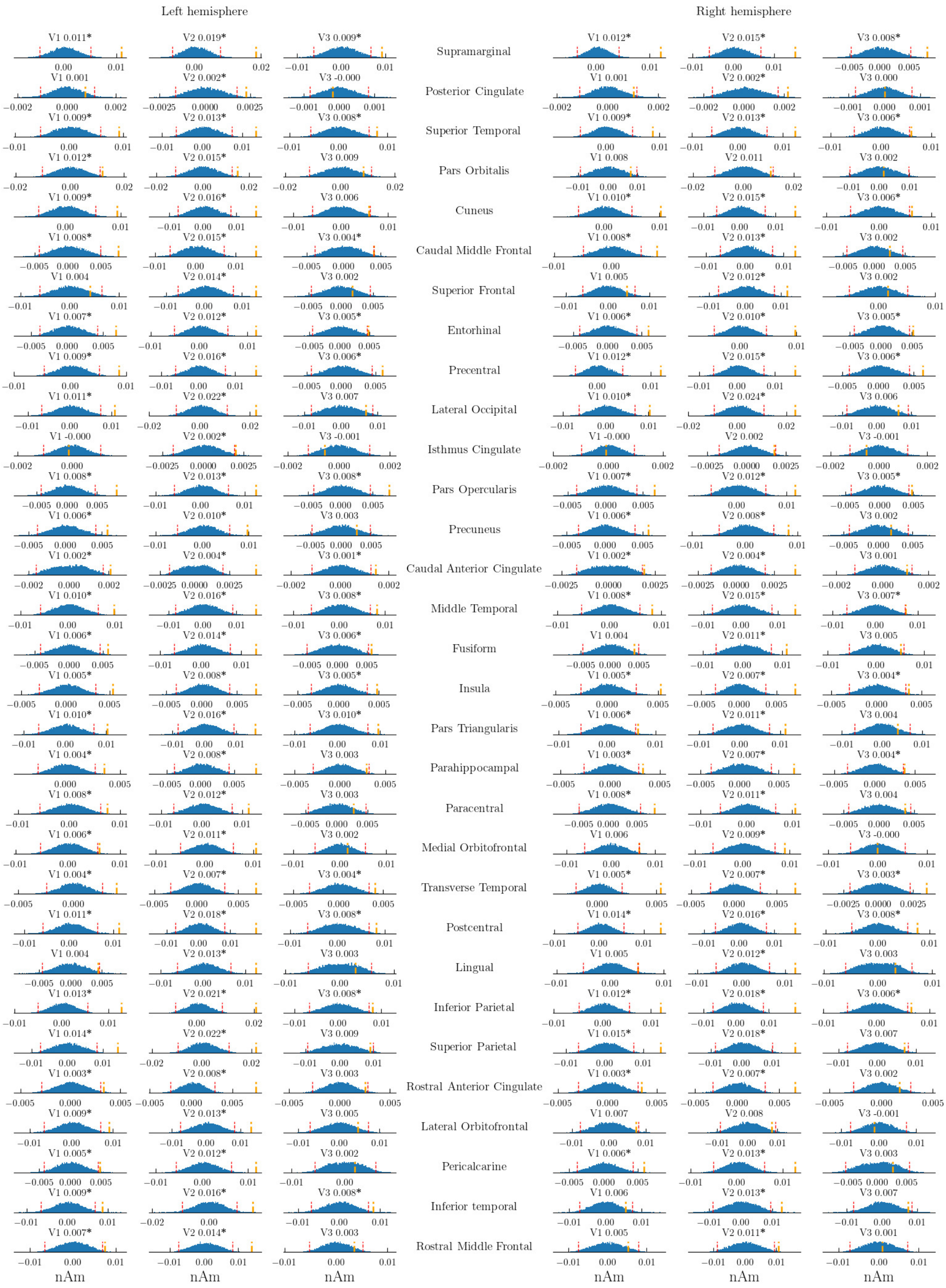

Figure S13. Between-region permutation tests grouped hemisphere.
